# A structural basis for antibody-mediated neutralization of Nipah virus reveals a site of vulnerability at the fusion glycoprotein apex

**DOI:** 10.1073/pnas.1912503116

**Published:** 2019-11-25

**Authors:** Victoria A. Avanzato, Kasopefoluwa Y. Oguntuyo, Marina Escalera-Zamudio, Bernardo Gutierrez, Michael Golden, Sergei L. Kosakovsky Pond, Rhys Pryce, Thomas S. Walter, Jeffrey Seow, Katie J. Doores, Oliver G. Pybus, Vincent J. Munster, Benhur Lee, Thomas A. Bowden

**Affiliations:** ^a^Division of Structural Biology, Wellcome Center for Human Genetics, University of Oxford, OX3 7BN Oxford, United Kingdom;; ^b^Laboratory of Virology, National Institute of Allergy and Infectious Diseases, National Institutes of Health, Hamilton, MT 59840;; ^c^Department of Microbiology, Icahn School of Medicine at Mount Sinai, New York, NY 10029;; ^d^Department of Zoology, Oxford University, OX1 3PS Oxford, United Kingdom;; ^e^Institute for Genomics and Evolutionary Medicine, Temple University, Philadelphia, PA 19122;; ^f^Department of Infectious Diseases, King’s College London, Guy’s Hospital, SE1 9RT London, United Kingdom;; ^g^Global Virus Network (GVN) Center of Excellence, Center for Virology, Icahn School of Medicine at Mount Sinai, New York, NY 10029

**Keywords:** henipavirus, glycoprotein, structure, antibody response, viral fusion

## Abstract

Nipah virus (NiV) is a highly pathogenic paramyxovirus that causes frequent outbreaks of severe neurologic and respiratory disease in humans with high case fatality rates. The 2 glycoproteins displayed on the surface of the virus, NiV-G and NiV-F, mediate host-cell attachment and membrane fusion, respectively, and are targets of the host antibody response. Here, we provide a molecular basis for neutralization of NiV through antibody-mediated targeting of NiV-F. Structural characterization of a neutralizing antibody (nAb) in complex with trimeric prefusion NiV-F reveals an epitope at the membrane-distal domain III (DIII) of the molecule, a region that undergoes substantial refolding during host-cell entry. The epitope of this monoclonal antibody (mAb66) is primarily protein-specific and we observe that glycosylation at the periphery of the interface likely does not inhibit mAb66 binding to NiV-F. Further characterization reveals that a Hendra virus-F–specific nAb (mAb36) and many antibodies in an antihenipavirus-F polyclonal antibody mixture (pAb835) also target this region of the molecule. Integrated with previously reported paramyxovirus F−nAb structures, these data support a model whereby the membrane-distal region of the F protein is targeted by the antibody-mediated immune response across henipaviruses. Notably, our domain-specific sequence analysis reveals no evidence of selective pressure at this region of the molecule, suggestive that functional constraints prevent immune-driven sequence variation. Combined, our data reveal the membrane-distal region of NiV-F as a site of vulnerability on the NiV surface.

## Significance

Despite causing regular outbreaks with high case fatality rates, there are currently no licensed vaccines or therapeutics for Nipah virus (NiV) infection. Here, we sought to determine the molecular basis for how the antibody response neutralizes NiV by targeting the surface-displayed fusion glycoprotein, NiV-F. Our structural study reveals a neutralizing antibody epitope at the membrane-distal portion of the prefusion trimeric NiV-F, which is well conserved across known NiV strains. Further structure–function analyses demonstrate that additional antibodies bind this region of the NiV-F apex, suggesting that this is an immunologically accessible site of vulnerability. This work reveals the membrane-distal regions of NiV-F and the F glycoprotein from closely related Hendra virus as attractive targets for antiviral and vaccine development.

The prototypic henipaviruses (HNVs), Hendra and Nipah virus, are highly pathogenic paramyxoviruses that have the potential to cause severe neurologic and respiratory disease in humans (1). Hendra virus (HeV) emerged in Australia in 1994, causing fatal disease in humans and horses, and continues to cause sporadic outbreaks (2, 3). Nipah virus (NiV) emerged in 1998 to 1999, causing an outbreak of severe encephalitis among pig farmers in Malaysia and Singapore, with a case fatality rate of 40% (4, 5). Since 2001, Bangladesh and India have also experienced regular NiV outbreaks (6–8), with the recent 2018 NiV outbreak in Kerala, India, exhibiting a 91% case fatality rate (9). Both HNVs circulate in reservoir host *Pteropus* bat populations (10, 11) and can cause spillover events into human populations through amplifying hosts, such as pigs and horses, or directly from bats, principally through the consumption of contaminated date palm sap (8). More alarmingly, human-to-human transmission has been documented during NiV spillover events in Bangladesh and India (8).

The enveloped surface of HNVs displays 2 viral glycoproteins, a receptor binding protein (G) and fusion glycoprotein (F), which mediate receptor attachment and membrane fusion, respectively (12–14). The HNV-F proteins are highly conserved, with 88% sequence identity between NiV-F and HeV-F, and 99% between different strains of NiV-F. HNV-F is a trimeric class I fusion protein that, similar to other paramyxoviral fusion proteins, consists of 3 domains (DI, DII, and DIII) in the globular head, followed by a C-terminal stalk, a transmembrane (TM) region, and a cytoplasmic tail (13, 15, 16). Two heptad-repeats (HR) are also present, HRA (heptad repeat A) in DIII and HRB (heptad repeat B) in the stalk. A cathepsin cleavage site and the hydrophobic fusion peptide are located within the DIII domain. Maturation of HNV-F occurs upon cathepsin-L–mediated cleavage of the precursor, F_0_, into 2 disulphide-linked components, F_1_ and F_2_ (17–19).

Although the structural basis of the interaction remains to be determined, G-mediated recognition of host cell-displayed ephrin receptors triggers HNV-F, initiating a cascade of conformational changes that results in the fusion of the viral and host cell membranes (20–26). Previous structural investigations have revealed that paramyxoviral F proteins undergo significant conformational rearrangement during membrane fusion, transitioning from a metastable prefusion to the more thermodynamically stable postfusion conformation. This process involves refolding of the DIII domain, allowing the insertion of the hydrophobic fusion peptide into the host-cell membrane, and reassembly of HRA and HRB to form the 6 helix bundle (6HB) postfusion state, which drives the merger of the virus and host cell membranes (15, 27–29).

As the sole proteinaceous antigens on the HNV surface, HNV-F and -G are principal targets of the host antibody response (30). Although no licensed vaccines or therapeutics for NiV currently exist, several experimental vaccine candidates that aim to elicit neutralizing antibody responses targeting these glycoproteins have shown promise. Various recombinant and attenuated vaccines expressing HNV-F and -G have been shown to be protective in hamster and African green monkey models (31–37). Additionally, several in vivo studies have demonstrated that treatment with monoclonal antibodies or passive transfer of antibodies targeting the F and G glycoproteins offers protection in hamsters, ferrets, and African green monkeys (31, 38–40), suggesting that neutralizing antibodies against the surface F and G glycoproteins are beneficial in combatting infection. The structure of a potent NiV and HeV cross-reactive nAb (m102.3) bound to HeV-G shows that the mechanism of neutralization involves occlusion of the receptor-binding site (41).

To further understand the molecular basis for antibody-mediated targeting and neutralization of HNVs, we determined the structure of an immunization-derived neutralizing monoclonal antibody (nAb) in complex with prefusion NiV-F. Our structure reveals that the nAb recognizes a predominantly protein-specific epitope on the membrane-distal DIII domain of the prefusion NiV-F trimer. Our integrated structural and functional study supports this immunologically accessible region as a site of vulnerability across NiV-F and HeV-F, and also provides a rationale for the conservation of certain N-glycan sites near this epitope. The high level of sequence conservation at this epitope further delineates this region as an attractive target for the development of henipaviral vaccines and therapeutics.

## Results

### Structural Characterization of NiV-F in Complex with Fab66.

mAb66 is a monoclonal antibody (mAb) that neutralizes NiV through recognition of NiV-F and was derived by DNA immunization of rabbits with expression plasmids encoding NiV-M, codon optimized NiV-F and NiV-G, and soluble NiV-G derived from the reference Malaysia strain (*SI Appendix*, Table S1) (42, 43). mAb66 neutralizes NiV-F/G mediated viral entry and fusion with an IC_50_ of <1 μg/mL (42). To determine the molecular basis for mAb66-mediated neutralization, we rescued the sequence of the corresponding Fab (fragment antigen binding) fragment of mAb66 (Fab66) (*Materials and Methods* and *SI Appendix*, Figs. S1 and S2), and subjected a recombinantly produced and deglycosylated NiV-F–Fab66 complex to crystallographic analysis (*Materials and Methods* and *SI Appendix*, Fig. S3). The crystal structure of NiV-F–Fab66 was solved to 3.2 Å resolution using the structures of NiV-F in the prefusion conformation [PDB ID code 5EVM (44)] and a rabbit Fab fragment [PDB ID code 4JO1 (45)] as molecular replacement search models (*SI Appendix*, Table S2).

**Fig. 1.**
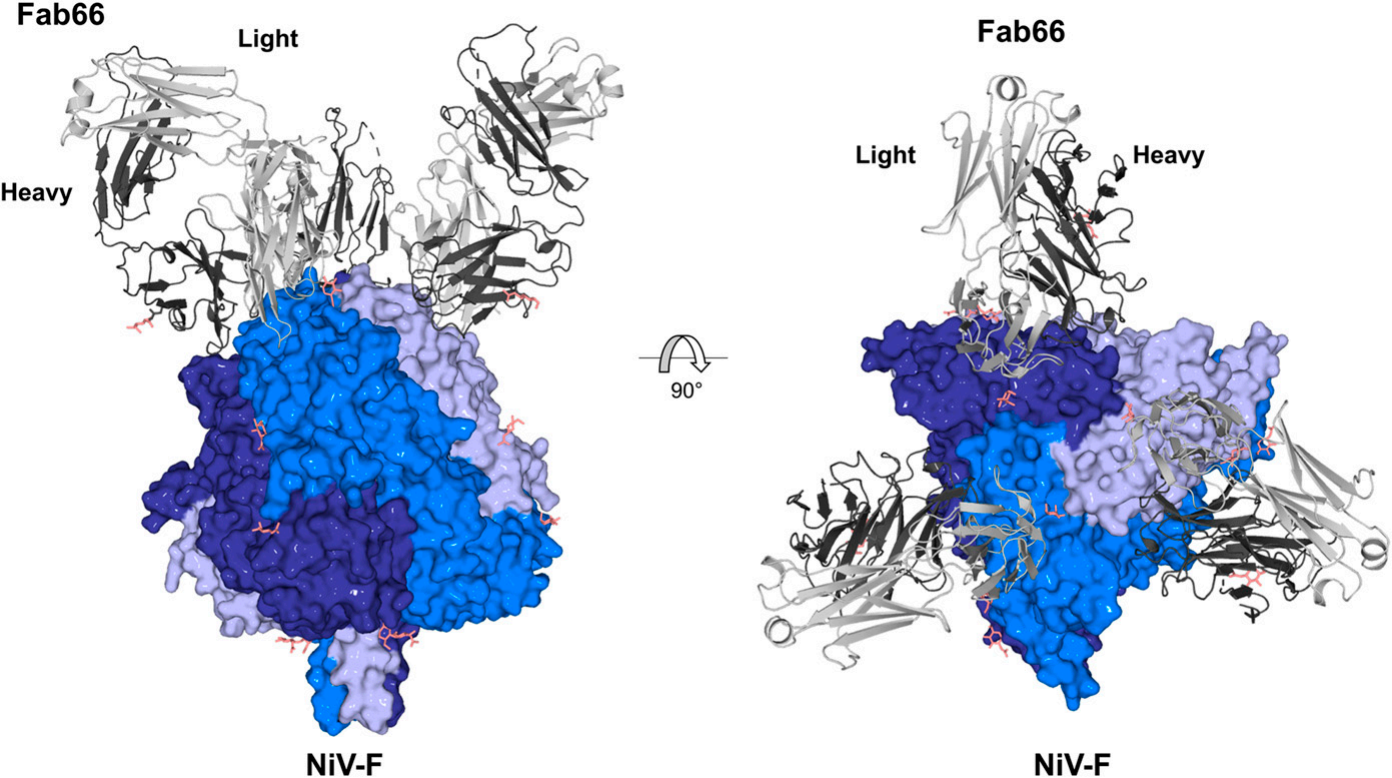
Crystal structure of the NiV-F−Fab66 complex. Side and top views show Fab66 bound to an epitope near the apex of the prefusion, uncleaved NiV-F trimer. Each Fab66 molecule binds to a single F protomer in the trimer. The NiV-F trimer is shown as surface with each protomer in the trimer colored a different shade of blue. Fab66 is shown as cartoon and the light and heavy chains are shown in light and dark gray, respectively. *N*-linked glycans are depicted as sticks and colored salmon.

Structural analysis revealed a crystallographic asymmetric unit containing a single NiV-F protomer bound to 1 Fab66. The trimeric biological assembly of the NiV-F–Fab66 complex, with 1 Fab molecule bound to each protomer of the trimer, is formed about a crystallographic symmetry axis (Fig. 1). Consistent with previously reported prefusion NiV-F, HeV-F, and PIV5-F (human parainfluenza virus 5) structures, Fab66-bound NiV-F adopts an uncleaved, prefusion conformation composed of domains DI, DII, DIII, and the HRB stalk (*SI Appendix*, Fig. S4) (15, 27, 44, 46). Fab66-bound NiV-F closely resembles the previously reported unbound, prefusion, and uncleaved structure of NiV-F [PDB ID code 5EVM (44); 0.7 Å root-mean-square-deviation (rmsd) over 1,322 equivalent Cα positions]. While structural overlay analysis indicates that Fab66 does not induce major conformational rearrangements to the molecule (*SI Appendix*, Fig. S5), we observe some local differences between Fab66-bound and unbound structures. Regions that exhibit structural variability (rmsd > 1.0 Å) are primarily located in solvent-exposed loops distal from the Fab66 binding site. However, residues Ser272−Ser273 and Ile190−Thr195 are included in the Fab66 epitope and present subtly different conformations, indicative of inherent flexibility or potentially small structural changes induced by Fab66 binding (*SI Appendix*, Fig. S5). Additionally, loop residues Leu104−Gly112, which encompass the cathepsin-L cleavage site (R109−L110), exist in a different conformation, consistent with the hypothesis that flexibility in this region may facilitate enzymatic cleavage into F_1_ and F_2_ (17–19, 44, 46).

### Fab66 Targets a Predominantly Protein-Specific Epitope.

**Fig. 2.**
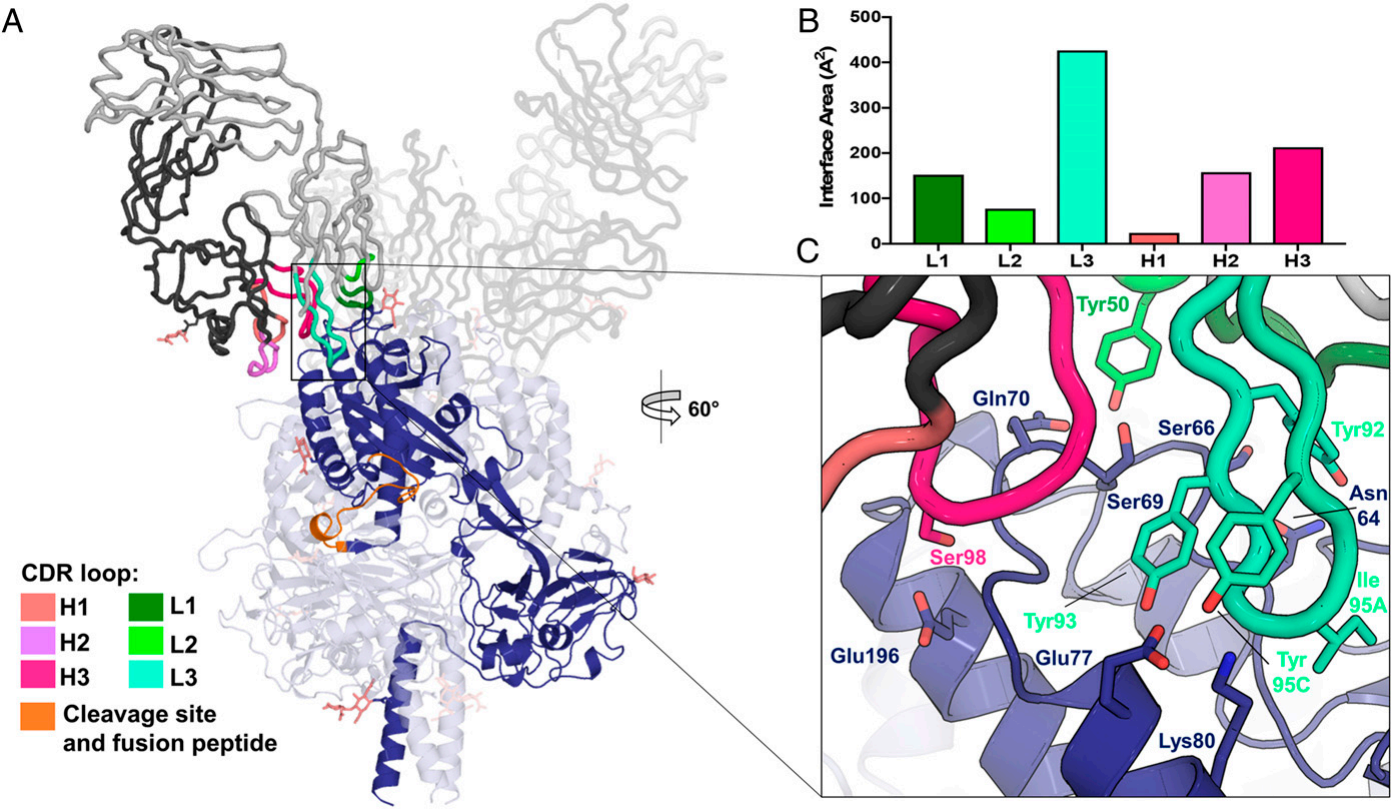
The NiV-F−Fab66 interaction is dominated by CDR L3. (*A*) A single NiV-F protomer and Fab66 are highlighted for clarity, where the F protomer is shown as a dark blue cartoon, Fab66 is shown as a gray cartoon tube, and the CDR loops are colored in shades of pink (heavy) and green (light), as indicated in the figure legend. The residues comprising the cleavage site and fusion peptide are shown in orange (V105−I122). *N*-linked glycans are depicted as sticks and colored salmon. (*B*) Contributions of each Fab66 CDR loop to the proteinaceous interface, calculated by the PDBePISA server (48), measured as interface area in Angstroms squared. (*C*) A close-up view of the interface between Fab66 and NiV-F. Side chains participating in intermolecular hydrogen bonds, as identified by the PDBePISA server (48), are shown as sticks. Residue Ile95A on CDR L3, the most buried residue in the complex, is also shown. CDR loop H2, though participating in important contacts, is not shown here and a detailed view on the interface can be found in *SI Appendix*, Fig. S7*B*.

Fab66 recognizes an epitope within DIII of NiV-F (Figs. 1 and 2 and *SI Appendix*, Fig. S4), with residues included within the range of Lys60–Lys80 forming the majority of the binding site. Interactions mediated by both the heavy and light chains comprise the ∼900 Å^2^ interface (Fig. 2*B* and *SI Appendix*, Fig. S6), where complementarity-determining regions (CDRs) from the light chain produce a larger footprint on the NiV-F surface (interface area of ∼575 Å^2^) than those from the heavy chain (∼350 Å^2^ interface area) (Fig. 2*B*). Indeed, while several important contacts are made by CDR loops L1, L2, H2, and H3 (Fig. 2 *B* and *C* and *SI Appendix*, Fig. S7), CDR L3 appears to dominate the interaction through extension of a 13-amino acid loop into a shallow depression flanked by 2 helices and an apical loop on the NiV-F surface (Fig. 2 *A* and *C*). Several tyrosine residues appear key for the interaction at this site, with the side chains of Tyr93 and Tyr95C in CDR L3 [Chothia numbering scheme (47)] forming hydrogen bonds with the side chains of Glu77 and Lys80 on NiV-F, and Tyr92 forming hydrogen bonds with the side chain of Asn64 (Fig. 2*C*). Furthermore, CDR L3 residue Ser94 also contributes to the interface through main-chain hydrogen bonding interactions between the carbonyl of Ser94 and the amine of Val65 on NiV-F. At the tip of CDR L3, the hydrophobic side chain of Ile95A buries into the NiV-F surface and has the highest buried surface area of any residue in the complex [calculated by the PDBePISA server (48)] (Fig. 2*C*). In addition to the CDR L3 contacts, hydrogen bond and hydrophobic interactions are also formed between the side chain of CDR L2 residue Tyr50 with NiV-F residues Ser66, Asn67, Ser69, and Gln70. The highly buried residue Ile30 on CDR L1 forms a backbone hydrogen bond with the side chain of residue Ser66 on NiV-F and also contributes to hydrophobic interactions with residues in the surrounding area (Fig. 2*C* and *SI Appendix*, Fig. S7*A*). On the heavy chain, CDR H2 residues Thr52A, Asn53, and Thr56 form extensive contacts with NiV-F residues Gly73, Ser74, and Glu77, while residues Ser98 and Gly99 on CDR H3 hydrogen bond with Glu196 and Gln70 on NiV-F. CDR H3 residues Trp100, Trp100B, and Tyr52 provide additional stabilizing interactions (*SI Appendix*, Fig. S7*B*).

**Fig. 3.**
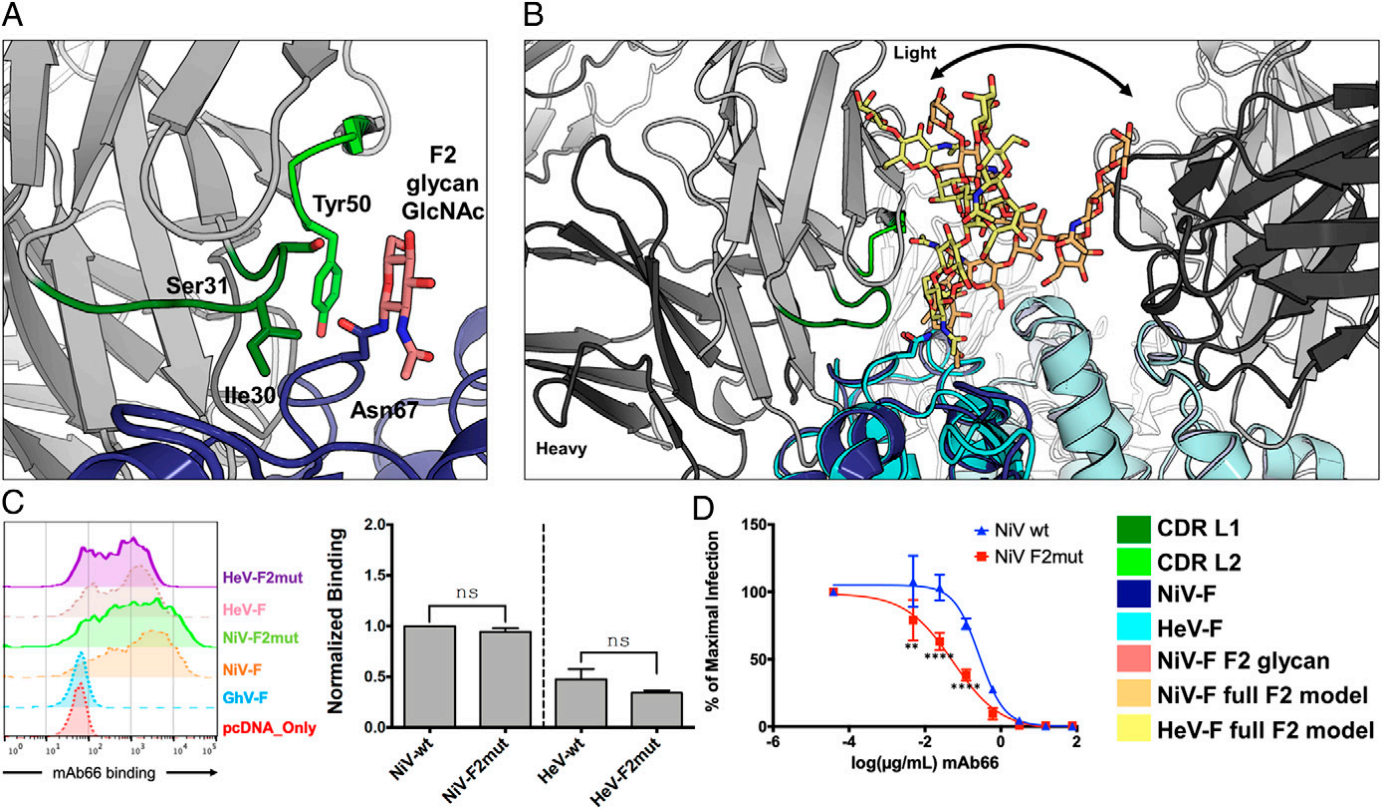
Glycosylation at the F2 N-glycan site on NiV-F. (*A*) The CDR L1 and L2 loops of Fab66 contact the GlcNAc residue of the F2 glycan site (Asn67) on NiV-F. Fab66 is shown as a cartoon tube (gray) and NiV-F is rendered as a cartoon (blue). CDR L1 and L2 are colored green, with the side chains of residues interacting with the GlcNAc (Ile30 and Ser31 of CDR L1 and Tyr50 of CDR L2) shown as sticks [calculated by PISA server (48)]. The corresponding Asn67 residue side chain is also shown as sticks and colored blue. (*B*) Modeling of a full-length complex *N*-linked glycan onto the F2 glycan site shows minimal steric hindrance to mAb66 binding. The structure of HeV-F [PDB ID code 5EJB (46), cyan] was aligned to NiV-F (dark blue). The full-length complex glycan chain from the structure PDB ID code 4BYH (80) was modeled onto the F2 glycan site on both NiV-F and HeV-F by aligning the first GlcNAc residues to the conformation observed in the crystal structures. The full-length glycan modeled onto NiV-F is shown as light orange sticks and the full-length glycan modeled onto HeV-F is shown as yellow sticks. The different conformations observed reflect the intrinsic flexibility expected of glycans (represented by the black arrow) and show that certain glycan conformations at F2 may only subtly interfere with mAb66 recognition. (*C*) Representative FACS histogram plots showing binding of mAb66 to WT NiV-F, NiV-F2mut, and their HeV-F counterparts expressed on transiently transfected 293T cells (*Left*). Binding data are presented as a bar graph (*Right*) of the mean ± SE for 3 independent replicates. Binding was first normalized to the binding of 2 anti-HNV polyclonal sera (pAb2489 and pAb2490), which were prescreened for equivalent cross reactivity to NiV-F and HeV-F as well as to all of the mutants examined in this study (*Materials and Methods* and *SI Appendix*, Fig. S10). These binding values were then renormalized to WT NiV-F binding set to 1 (normalized binding, *y* axis). Statistical significance was determined by an ordinary 1-way ANOVA with Sidak’s correction for multiple comparisons (n.s., not significant, or *P* > 0.05). (*D*) mAb66 neutralization of NiV-F/G pseudotyped [VSV-ΔGRLuc] particle (NiVpp) infection on permissive U-87 MG glioblastoma cells. NiVpp bearing WT NiV-G and the indicated homologous WT or mutant NiV-F (*SI Appendix*, Fig. S11) were used to infect U87 cells in the presence of serial 5-fold dilution of mAb66 as described in *Materials and Methods*. Infections were performed using optimized virus inputs that will give reporter gene (*Renilla* luciferase, RLuc) outputs (relative light units) within the dynamic response range of the assay for all mutants tested (*SI Appendix*, Fig. S12). Data were analyzed using nonlinear regression, fitted using a variable slope model, and presented as a 4-parameter dose–response curve (GraphPad PRISM). The lowest value on the *x* axis (mAb), “media only,” is artificially set to constrain the level of maximum infection (*y* axis) in the absence of any mAb. Data points are mean ± SE for each neutralization curve performed in biological triplicates; each replicate comprising of technical duplicates. Statistical significance for the neutralization assay was tested with 2-way ANOVA with Dunnett’s correction for multiple comparison (**P < 0.01; ****P < 0.0001).

NiV-F encodes 5 putative *N*-linked glycosylation sites (F1 to F5), 4 of which have been shown to be occupied in the recombinant soluble protein (44, 49) and in NiV-F present on infectious NiV-F/G pseudotyped particles (NiVpp) (50, 51). Consistent with these previous observations, and the deglycosylation of our NiV-F with endoglycosidase F_1_ (52), we observe well-ordered electron density corresponding to an *N*-acetyl glucosamine (GlcNAc) moiety at 4 of the 5 glycosylation sites (sites F2 to F5) (*SI Appendix*, Fig. S8). The F1 site did not show any evidence for a glycan being present. Previous work has demonstrated that *N*-linked glycans on NiV-F play a role in protection from the neutralizing antibody response, particularly the glycans at the F2 and F5 sites (50). Interestingly, the highly conserved F2 glycan site (Asn67) (48) is located at the periphery of the Fab66 epitope, wherein the crystallographically observed GlcNAc forms contacts with residues Ile30, Ser31, and Tyr50 of CDR loops L1 and L2, which approach from the side of the glycan (Fig. 3*A*). Although our NiV-F was partially deglycosylated, modeling suggests that the complex glycan (51) extending from the F2 site would be unlikely to hinder the angle of approach by CDR loops L1 and L2 to the corresponding or neighboring NiV-F and HeV-F protomers, suggesting that mAb66 is able to access its proteinaceous epitope on the NiV-F surface despite the presence of the F2 glycan (Fig. 3*B*). Consistent with this model, removal of the F2 glycan site on the HNV-F surface (NiV-F2 and HeV-F2mut) did not significantly affect the binding affinity of mAb66 to NiV-F or HeV-F (Fig. 3*C*), although our binding data reinforced previous observations that mAb66 binds better to NiV-F than to HeV-F (42).

Interestingly, while mAb66 binding to both NiV-F2 and HeV-F2 mutants was unaffected, we note a significant increase in neutralizing potency of mAb66 against the NiV-F2mut in our infectious vesicular stomatitis virus (VSV)-based NiV-F/G pseudotyped particle (NiVpp) infection assay (Fig. 3*D* and Table 1). Furthermore, no differences in neutralization of the WT HNV-F and HNV-F2mut was observed using our anti–G-specific polyclonal antibody, pAb1187 (*SI Appendix*, Figs. S9–S12), suggestive that the F2 mutation is unlikely to affect the overall virus structure or the interactions between HNV-F and HNV-G. Thus, the observed neutralization difference may be a consequence of subtle variations in binding dynamics, potentially due to changes in accessibility from the minor clashes with certain glycan conformations (Fig. 3*B*). Indeed, the presence of NiV-G on infectious NiVpp, in the context of the dense array of glycoproteins on the virion, likely renders mAb66 more sensitive to potential glycan blockade. The next subsection on the membrane distal surface of NiV-F provides a more detailed explanation of our model for the immunological accessibility of the mAb66 epitope.

**Table 1.** Neutralizing potency of Mab66 against NIVpp and HeVpp bearing WT G and the indicated F mutants

	NiVpp, µg/mL	HeVpp, µg/mL
WT G/F	0.29	3.35
WT G/F2mut	0.05	3.81
WT G/F 70+74mut	2.97	1.45

**Fig. 4.**
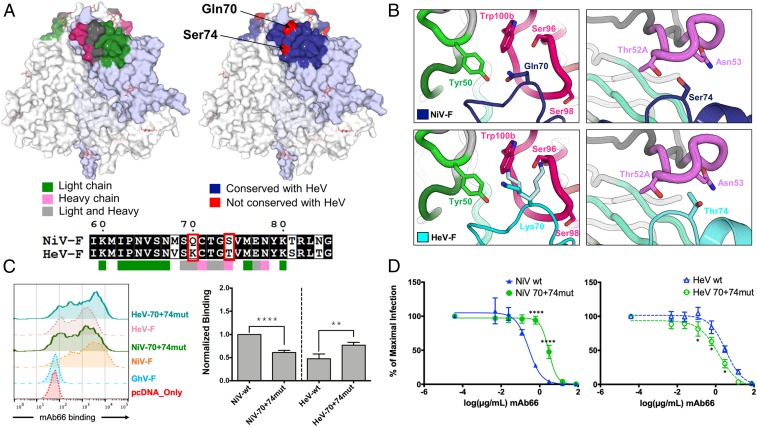
Differences between NiV-F and HeV-F at the Fab66 footprint. (*A*) Surface view of the NiV-F is shown in white, with 1 protomer shaded in light blue for clarity. (*Upper Left*) The residues in the binding interface are colored according to the Fab66 chain involved in the contact, with residues contacted by the light chain shown as green, heavy-chain contacts shown as pink, and residues contacted by both chains shown in gray. (*Lower*) A sequence alignment between NiV-F (Malaysia, AAV80428.1) and HeV-F (AEB21197.1), was generated by Multalin (81) and plotted by ESPript (82). Residues involved in the Fab66 interface are noted with colored boxes below the alignment. Residues that differ between NiV-F and HeV-F are outlined by a red box. (*Upper Right*) The entire binding footprint of Fab66 is shown as blue. The residues that differ between NiV-F and HeV-F within the epitope, Gln70 and Ser74, are shown in red and labeled. (*B*) To model Fab66 binding at residues 70 and 74 on NiV-F (*Upper*) and HeV-F (*Lower*), the HeV-F structure [PDB ID code 5EJB (46), cyan cartoon] was aligned to NiV-F (dark blue cartoon). (*Upper Left*) Representation of Gln70 on NiV-F (dark blue), which forms hydrogen bond contacts with Tyr50 (CDR L2, light green) and Ser98 (CDR H3, hot pink). (*Upper Right*) Representation of Ser74 on NiV-F (dark blue), which forms hydrogen bond contacts with residues Thr52A and Asn53 (CDR H2, pink) of Fab66. (*Lower Right*) The threonine at position 74 in HeV-F (cyan) does not show obvious clashes with CDR H2 of Fab66. (*Lower Left*) The lysine substitution in HeV at position 70 shows the lysine side chain as modeled in the crystal structure [PDB ID code 5EJB (46), cyan], as well as 2 rotamers (light cyan) that clash with residues Ser96 and Trp100B in CDR H3 (hot pink) of Fab66. (*C*) Representative FACS histogram plots showing binding of mAb66 to WT NiV-F, HeV-F and the indicated reciprocal double mutant (70+74mut), as described in the text (*Left*). Normalized mAb66 binding were analyzed and presented as bar graphs (*Right*). The experiment was performed and analyzed as in Fig. 3*C* (**P < 0.01; ****P < 0.0001). (*D*) mAb66 neutralization curves of NiVpp and HeVpp infection on permissive U87 glioblastoma cells were generated as detailed in Fig. 3*D*, with the exception of the different HNV-F mutants used here. Each neutralization curve was performed in biological triplicates comprising of technical duplicates per biological replicate. Data was analyzed as in Fig. 3*D* (*P < 0.05; ****P < 0.0001).

While the Fab66 epitope is highly conserved across all known NiV isolates (*SI Appendix*, Fig. S13), previous functional studies indicate that mAb66 binds better to NiV-F than HeV-F (Fig. 3*C*) (42). Interestingly, mapping of residues contacted by the heavy and light chains of Fab66 onto an amino acid sequence alignment of NiV-F and HeV-F reveals that only 2 residues differ between the 2 viruses at the binding interface (Fig. 4*A* and *SI Appendix*, Fig. S6), where Gln70 and Ser74 in NiV-F are replaced by Lys70 and Thr74, respectively, in all analyzed HeV-F sequences. Both Gln70 and Ser74 form extensive interactions with Fab66 (Fig. 4 *B*, *Upper*), with Gln70 exhibiting a high buried surface area within the complex [calculated by the PDBePISA server (48)]. The side chain of Ser74 forms hydrogen bonds with Thr52A and Asn53 of CDR H2 (Fig. 4 *B*, *Upper Right*), while the side chain and backbone of Gln70 form hydrogen bonds with Ser98 and Gly99 of CDR H3 and Tyr50 of CDR L2 (Fig. 4 *B*, *Upper Left*). Modeling Fab66 bound to HeV-F shows that the serine-to-threonine substitution at position 74 does not introduce any obvious steric clashes (Fig. 4 *B*, *Lower Right*); however, the glutamine-to-lysine substitution at position 70 introduces a larger, positively charged side chain (Fig. 4 *B*, *Lower Left*). Although several lysine side chain conformations can be accommodated in the model, certain side chain rotamers introduce clashes with residues in CDR H3, specifically Trp100B and Ser96, and may be less favorable for hydrogen bond formation (Fig. 4 *B*, *Lower Left*), which could result in the lower binding ability of mAb66 for HeV-F relative to NiV-F observed in vitro (Fig. 3*C*) (42). To test this hypothesis, we created a double K70Q + T74S HeV-F mutant, in which the 2 key residues at positions 70 and 74 were changed to their NiV counterparts (HeV-70+74mut). This minimal substitution (HeV-70+74mut) was sufficient to enhance mAb66 binding of HeV-F by ∼2-fold (Fig. 4*C*). Conversely, the reciprocal Q70K + S74T NiV-F double mutant (NiV-70+74mut) decreased mAb66 binding to NiV-F to the level seen for HeV-70+74mut (Fig. 4*C*). These differences in mAb66 binding to HeV-70+74mut and NiV-70+74mut were reflected in the increased and decreased sensitivity to mAb66 neutralization, relative to their cognate WT counterparts (Fig. 4*D* and Table 1), confirming that differences at these residues confer lower binding and neutralization potency of mAb66 for HeV compared to NiV, although additional local differences on the HeV-F and NiV-F protein surfaces may also contribute to these observations.

### The Membrane Distal Surface of NiV-F Is Immunologically Accessible yet Functionally and Evolutionarily Constrained.

**Fig. 5.**
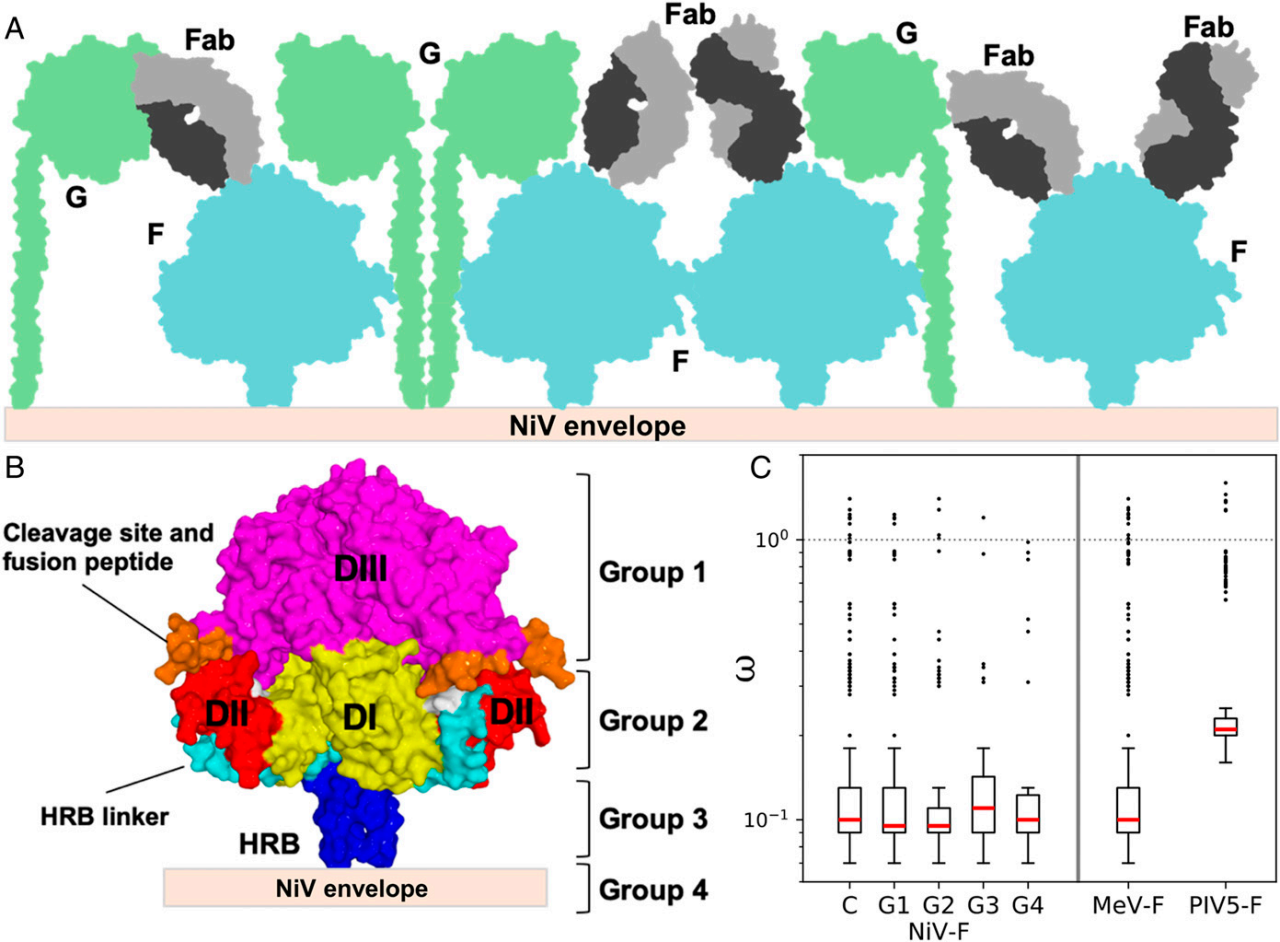
A model for immune accessible areas on NiV-F. (*A*) Schematic of the NiV surface, where NiV-F (blue) and NiV-G (green) associate and densely populate the viral envelope. Fabs (gray) are shown contacting potential immune accessible, membrane distal regions of NiV-F. (*B*) Diagram showing the functional domains of the NiV-F protein and the assignment of groups for the dN/dS analysis. Groups were assigned based on functional domain and distance from the viral membrane. Group 1: DIII; group 2: DI, DII, and the HRB linker; group 3: HRB; and group 4: TM domain. (*C*) Comparison of the ω estimates (*y* axis) across the complete fusion protein (termed “C”) of 3 paramyxoviruses (NiV-F, MeV-F, and PIV5-F), and across different functional groups of the NiV-F (G1 to G4, as defined in *B*). No evidence for positive diversifying selection was detected for any region of the different paramyxoviral F proteins analyzed, as all mean estimates fall below the threshold of ω > 1 (represented by the dotted line at 10^0^). The red lines represent the median values, the ends of each box represent the 75% confidence intervals, and the whiskers represent the 95% CIs. The outliers are values that lie beyond the 95% CIs and are represented by circles (see *SI Appendix*, Table S3).

Electron microscopy studies have previously demonstrated that the paramyxoviral envelope is decorated with a heterogeneous assembly of receptor-binding and fusion glycoproteins (53, 54). Consistent with the hypothesis that the dense population of these proteins may result in membrane distal regions of NiV-F being more accessible to the host immune response (Fig. 5*A*), we observe that Fab66 binds near the apex of the NiV-F globular head (Fig. 1). Similarly located epitopes have also been observed on the prefusion structures of PIV3-F (55).

To assess whether this exposed region may be subject to selective pressure, such as that imposed by the immune response, we performed dN/dS ratio (ω) estimation analysis to quantify potential positive selection on NiV-F using several different methods. We estimated and compared the ω distributions among each site for the entire protein, between buried and solvent exposed residues, and among domain-specific regions of the protein (Fig. 5 *B* and *C* and *SI Appendix*, Figs. S14 and S15 and Tables S3 and S4). For this purpose, the protein was divided into 4 groups (G1 to G4) based on the functional domains of the protein and their distance relative to the virus membrane (Fig. 5*B*). Group 1 corresponds to DIII, which is the most membrane-distal and exposed portion of the globular head, and is where the reported Fab66 epitope is located. The other structurally defined groups were DI, DII, and the HRB linker (group 2) in the lower half of the globular head, the HRB (group 3), and the TM domain (group 4) (Fig. 5*B*). Given that the diversity and number of sequences available for NiV is limited, we sought to increase the power of the analysis by extrapolating any patterns of diversifying selection found within the fusion proteins of measles (MeV) and PIV5, which share similar structural and functional features as NiV-F and for which there are more available sequences.

Using both gene and site-level dN/dS (ω) estimation methods and a branch-site unrestricted model to detect gene-level episodic diversification customized for our datasets (*Materials and Methods* and *SI Appendix*, *Supplementary Methods*), we found no evidence for positive selection acting on the complete NiV-F coding sequence, or for any of the structural groups defined, with no individual positively selected sites (Fig. 5*C* and *SI Appendix*, Fig. S14 and Tables S3 and S4). This includes the apex region of DIII, for which a mean ω of 0.35 was estimated (median ω = 0.11, 95% CI = [0.111 to 0.160]), and the complete Fab66 epitope, for which a mean ω = 0.158 was obtained (median ω = 0.11, 95% CI = [0.097 to 0.219]), respectively, revealing no evidence of selection. Similarly, we found no evidence for positive selection acting on the MeV-F and PIV5-F proteins using a comparative approach. The high degree of conservation at a sequence level within the exposed region of the paramyxoviral fusion protein suggests that strong functional and structural constraints are maintained through purifying selection, despite exposure to any existing pressure from the host immune response. This is further supported by the observation that the highest proportion of sites within the alignments are assigned to the strong conservation class (ω << 1) under both CODEML (56) and FUBAR (57) approaches (*SI Appendix*, Figs. S14 and S15), and that, despite low sequence similarity, several conserved functional and structural domains are observed for the F protein across distant paramyxoviruses (16). Combined with a relative lack of antigenic drift in paramyxoviruses (compared to other RNA viruses) (58) and the evidence indicating that the MeV envelope glycoproteins show low tolerance to amino acid insertions through a whole-genome transposon mutagenesis screen (59), these results are consistent with a model whereby paramyxoviral fusion glycoproteins are subject to strong functional constraints, which curb genetic variation, to sustain a highly conserved role.

**Fig. 6.**
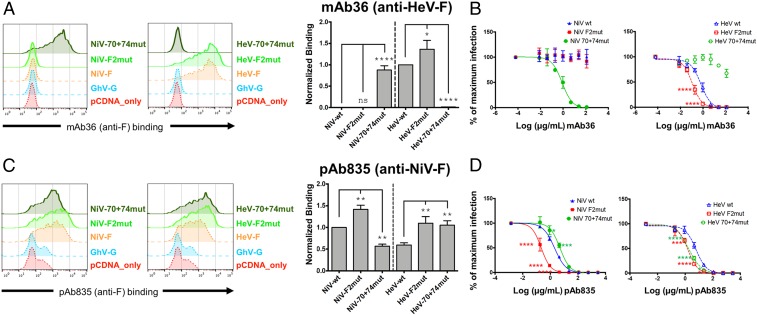
Anti-F specific monoclonal and polyclonal antibodies are sensitive to mutations at the apex of NiV-F and HeV-F. (*A* and *C*) Representative FACS histogram plots showing binding of mAb36 or pAb835 to WT NiV-F, HeV-F, the cognate HNV-F2 mutants (NiV-F2mut, HeV-F2mut), or the reciprocal double mutants (NiV-70+74mut, HeV-70+74mut) as described in the text (*Left*). Normalized binding analyzed and presented as a bar graph as described for Fig. 3*C* (*Right*, mean ± SE, *n* = 3). Binding values were normalized to HeV-F for mAb36 and to NiV-F for pAb835. (*B* and *D*) Neutralization curves of NiVpp (*Left*) and HeVpp (*Right*) infection using WT, F2mut, and the cognate 70+74mut, were generated and analyzed as described in Fig. 3*D*. Each curve was performed in biological triplicates comprised of technical duplicates per biological replicate. Data points shown are mean ± SE. Statistical significance was determined as described in in Fig. 3*D* legend (*P ≤ 0.05, **P ≤ 0.01, ***P ≤ 0.001; ****P ≤ 0.0001). See *SI Appendix*, Table S1 for documentation of anti-F and anti-G specific polyclonal antibody specificities. As a control, neutralizations were performed with an anti-G specific polyclonal and revealed no differences in neutralization of the WT and mutant F constructs (*SI Appendix*, Fig. S9).

To determine if the evidently immunologically accessible apex region might also be targeted by additional antibodies, we isolated and preliminarily characterized another rabbit nAb, termed mAb36, from a DNA immunization scheme that used only HeV envelope glycoproteins (*SI Appendix*, Table S1), but generated in a similar fashion to mAb66. As shown in Fig. 6*A*, mAb36 specifically binds HeV-F but not NiV-F. Remarkably, a switch of the same 2 residues at positions 70 and 74 in NiV-F to its HeV-F counterparts (Q70K+S74T; NiV-70+74mut) was sufficient to confer mAb36 binding equivalent to WT HeV-F binding, as well as neutralization (Fig. 6 *A* and *B*). Conversely, the reciprocal switch in HeV-F to its NiV-F counterparts (K70Q+T74S; HeV-70+74mut) completely abrogated mAb36 binding and neutralization to HeV-F. Furthermore, mAb36 exhibited increased binding and neutralization to the HeV-F2mut (Fig. 6 *A* and *B*). Thus, it appears that an HeV-F–specific mAb, from a completely different immunization scheme, targets a nearby epitope that sterically overlaps with the footprint of mAb66 at the membrane distal apex portion of the HNV-F trimer (the tip of DIII). To provide further evidence supporting this region as a commonly targeted site of immune vulnerability, we tested our polyclonal anti–NiV-F sera, pAb835 (*SI Appendix*, Figs. S16 and S17), for binding and neutralization against the 70+74 and F2 mutants. Also remarkably, pAb835 exhibited reduced binding and neutralization to the NiV-70+74mut and increased binding and neutralization to the HeV-70+74mut, with respect to the WT HNV-F controls, suggestive that many antibodies within the polyclonal serum bind within this region. Similarly, pAb835 also exhibited increased binding and neutralization to both NiV-F2 and HeV-F2 mutants (Fig. 6 *C* and *D*). As these mutations located at the apex of DIII are capable of affecting binding of pAb835, these results provide additional support for the hypothesis that this region is immunologically accessible and may be a common target of the host antibody response.

## Discussion

NiV continues to cause highly fatal outbreaks in Southeast Asia, highlighting the need for research to both further understand how the immune system successfully combats this pathogen, and to develop effective vaccines and therapeutics. Although the immunological factors that determine the outcome of NiV infection in humans and other hosts are yet to be thoroughly characterized, experimental vaccination and in vivo protection studies using therapeutic or convalescent-derived antibodies (31–41) suggest that the development of a strong neutralizing antibody response against NiV-F and NiV-G is a viable strategy for combatting the virus (30).

Here, we provide molecular-level insights into how the neutralizing antibody response can target NiV-F. Our structural analysis of the NiV-F−Fab66 complex revealed that the nAb binds to the prefusion conformation of NiV-F at the tip of the DIII domain of the molecule in a predominantly protein-specific interaction that is dominated by CDR loop 3 of the light chain (Figs. 1 and 2). The epitope is highly conserved between NiV Malaysia and Bangladesh strains (*SI Appendix*, Fig. S13), and substitutions of only 2 residues are required to enhance binding and neutralization of HeV-F (Fig. 4 *C* and *D*). Interestingly, we note that the DIII domain of NiV-F dramatically refolds during the fusogenic rearrangements that drive the merger of the viral and host-cell membranes (15, 27). Furthermore, structure-based mapping using a model of PIV3-F in the postfusion conformation reveals that the equivalent residues constituting the Fab66 epitope become disrupted upon 6HB formation (*SI Appendix*, Fig. S18). While there are several potential mechanisms by which a fusion protein-specific antibody may interrupt the fusion cascade, this observation leads us to hypothesize that mAb66 may interfere with the transition of NiV-F to the postfusion conformation, although further experimental evidence is needed to confirm the mechanism.

While the interactions and higher-order assembly of paramyxovirus fusion and receptor-binding glycoproteins remain to be determined, several structural studies have reported the paramyxoviral envelope surface to be densely populated by these glycoproteins (53, 54). Consistent with a model whereby the membrane-distal regions of F and G are the most immune accessible, the Fab66 epitope localizes toward the apex of NiV-F, a region distal from the virus membrane (Figs. 4*A* and 5*B*). Furthermore, our epitope mapping analysis reveals that a spatially similar site is targeted on HeV-F by the HeV-specific mAb36 and that anti-F polyclonal sera (pAb835) binding is affected by residue 70+74 mutations and F2 mutations at the DIII apex (Fig. 6 and *SI Appendix*, Fig. S17), supporting the hypothesis that this region on HNV-F is a common target for antibodies arising from infection and immunization, more broadly.

To assess whether this antigenic membrane-distal region is subject to selective pressure from the host immune system, we subjected reported paramyxoviral fusion glycoprotein sequences to a structure-based, domain-specific analyses for detecting diversifying positive selection. Our analysis reveals that this region of the molecule undergoes limited adaptive genetic change not only in NiV-F, but also in more distantly related paramyxoviral MeV-F and PIV5-F (Fig. 5*C* and *SI Appendix*, Figs. S14 and S15 and Tables S3 and S4). Although it has been proposed that paramyxoviral F proteins can be activated differently by attachment proteins H, HN, or G, the high level of sequence conservation is independent of these models of fusion/attachment protein assembly and activation (29). These results contrast recent studies on the Lassa virus glycoprotein precursor and Ebola virus glycoprotein, which show that residues with the highest level of nonsynonymous substitutions map to exposed regions of the glycoprotein surface (60, 61). The limited level of genetic variation and lack of signal for diversifying positive selection among any functional domain of the F protein, the evidence for purifying selection (Fig. 5*C* and *SI Appendix*, Tables S3 and S4 and Figs. S14 and S15), and the conserved structural folding pattern observed among distant paramyxoviral F proteins, likely reflects the existence of functional constraints on the molecule and highlights the necessity of membrane fusion for the virus to establish infection. Such constraints imposed on the molecule appear to outweigh any selective pressure that may be imposed by the antibody-mediated immune response arising during infection, though it is also possible that functional constraints exist in the absence of selective pressures. Indeed, although we found no evidence for individual sites that may be under selective pressure and global dN/dS estimates yielded values <<1, given the limited number and diversity of NiV-F sequences currently available, it is possible that the current dN/dS estimation methods are not able to detect signatures for positive selection. The strict requirement to maintain this functionality establishes this as a site of vulnerability on the NiV surface and thus an attractive target for vaccine and monoclonal antibody development.

The sustained threat NiV and HeV pose to human health, combined with the growing diversity of newly discovered HNVs and an extensive geographic distribution (62), highlights the need to define vulnerable epitopes on the HNV surface and to develop effective therapeutic strategies to prevent and respond to infection. This work provides an initial model for how the host antibody response can neutralize NiV by targeting the fusion glycoprotein at a membrane-distal epitope. The observation that this region of the fusion glycoprotein appears to be also targeted on HeV supports the apex of DIII as a site of vulnerability across HNV-Fs, more broadly. Thus, this work offers a structure-based rationale for the design of therapeutics and vaccines targeting HNV-F. Further assessment of the in vivo activity of anti-F mAbs targeting the DIII domain of HNV-F, both alone and in combination with mAbs that target spatially distinct epitopes on the HNV surface (e.g., HNV-G or other HNV-F epitopes), constitutes a logical next step in guiding the development of biologic countermeasures against these highly lethal pathogens.

## Materials and Methods

### Sequencing and Cloning of mAb66 Variable Regions from Hybridoma Cell Line.

Hybridomas were cultured in medium E (Clonacell) at 37 °C with 5% humidity. When cells achieved appropriate density, total RNA was extracted using the Qiagen RNeasy mini kit, per the manufacturer’s protocol. cDNA was generated using the Invitrogen SuperScript IV First Strand Synthesis System using random hexamers following the manufacturer’s protocol. The variable regions of heavy and κ chains were PCR-amplified using previously described rabbit (63) primers and PCR conditions. PCR products were purified and cloned into an expression plasmid (63, 64) adapted from the pFUSE-rIgG-Fc and pFUSE2-CLIg-rK1 vectors (InvivoGen) using the Gibson Assembly Master Mix (New England Biolabs) under ampicillin selection following the manufacturer’s protocol. Antibody heavy and light plasmids were cotransfected at a 1:1 ratio into HEK293F cells (ThermoFisher) using PEI Max 40K (linear polyethylenimine hydrochloride, Polysciences). Antibody supernatant was harvested 4 d following transfection and purified using protein G affinity chromatography following the manufacturers protocol (GE Healthcare).

### Antibody Binding to Recombinant Soluble NiV-F.

High-binding ELISA 96 half-well microplates (Corning) were coated with purified NiV-F (25 μL, 3 μg/mL in PBS) overnight at 4 °C. Plates were washed 5 times with PBS containing 0.05% Tween20 (PBS-T) and blocked with blocking buffer (5% nonfat milk in PBS-T) for 1 h at room temperature. The blocking buffer was removed and serial diluted antibody (mAb66 purified from hybridoma cells and expressed from cloned mAb66) (starting at 50 μg/mL, 1:5 dilution in blocking buffer) was added for 2 h at room temperature. Plates were washed 5 times with PBS-T. Secondary antibody (goat anti-Rabbit IgG F(ab')_2_, AP conjugate, Invitrogen, 1:1,000) was added for 1 h and plates were washed, as described above. The p-nitrophenyl phosphate substrate (Sigma) was added to detect binding and OD were measured at 405 nm (*SI Appendix*, Fig. S1).

### Protein Production.

The ectodomain of NiV-F (residues G26**−**D482) was cloned into the pHLsec vector (65) containing the C-terminal GCNt trimerization motif (MKQIEDKIEEILSKIYHIENEIARIKKLIGE) in place of the TM and cytoplasmic domains, as previously described (37, 49). The protein was expressed via transient transfection of HEK293T cells in the presence of the α-mannosidase I inhibitor kifunensine (52, 65). Cell supernatant was harvested 5 d posttransfection, clarified by centrifugation, and diafiltrated against 10 mM Tris pH 8.0, 150 mM NaCl using an AKTA Flux system (GE Healthcare). The protein was further purified by Ni-NTA immobilized metal-affinity chromatography (IMAC) using His-Trap HP columns (GE Healthcare), followed by size-exclusion chromatography (SEC) using a Superose 6 Increase 10/300 column (GE Healthcare) equilibrated with 10 mM Tris pH 8.0, 150 mM NaCl. For Fab66 production, Fab heavy and κ chains were synthesized by Geneart (Life Technologies) and cloned into the pHLsec vector (65). Only the heavy-chain construct contained a C-terminal hexahistidine tag. The heavy and κ plasmids were cotransfected in HEK293T cells in a 1:1 mass ratio and purified by IMAC and SEC, as described above.

### Crystallization and Structure Determination.

Prior to crystallization, recombinant Fab66 and NiV-F were mixed in a 3.3:1 molar ratio (Fab66 to NiV-F) and incubated at room temperature for 1 h. The excess Fab was removed via SEC, as described above. Prior to crystallization, the complex was partially deglycosylated with endoglycosidase F_1_ (52), repurified by SEC, and concentrated to 5.0 mg/mL. Crystallization screens were set up using the sitting-drop vapor-diffusion method, using 100 nL protein complex plus 100 nL precipitant, as previously described (66). Optimized crystals used for data collection were grown at room temperature in a precipitant containing 0.094 M Tris pH 8.0 and 3.7 M NaCl. Crystals were cryoprotected by immersion in precipitant supplemented with 25% glycerol (vol/vol) and flash-frozen in liquid nitrogen.

Diffraction data for the NiV-F−Fab66 complex were collected on the I03 beamline at Diamond Light Source (Didcot). Data were indexed, integrated, and scaled using XIA2 DIALS (67) and the high-resolution cutoff was determined by assessment of I/Iσ and CC_1/2_. The structure of the complex was solved by molecular replacement in PHASER (68) using the structures of the prefusion NiV-F [PDB ID code 5EVM (44)] and a rabbit Fab [PDB ID code 4JO1 (45)] as search models. Model building was performed using real-space refinement in COOT (69) and refinement was performed using REFMAC5 in the CCP4 suite (70, 71) and PHENIX refine with TLS parameterization (72). COOT and Molprobity (73) were used to validate the final models and used alongside *R*_work_ and *R*_free_ to monitor the quality of the models. The Pymol Molecular Graphics System (https://www.schrodinger.com/pymol) was used to generate the structural models presented in the figures. The atomic coordinates and structure factors of the NiV-F-Fab66 complex were deposited in the Protein Data Bank (PDB), PDB code 6T3F (74).

### Cell Surface Expression and Antibody Binding Assays.

HEK293T and U87 cells were grown in DMEM supplemented with 10% FBS. NiV and HeV F and G glycoproteins were codon optimized and contained a C-terminal AU1 or HA tag, respectively. All mutant F constructs were cloned into a pCAGG vector by using overlap PCR. *N*-linked glycosylation sites were removed by making a conservative asparagine-to-glutamine change, as previously described (50). Cell surface expression of WT and mutant F glycoproteins was assessed by transfection into 293T cells with PEI (Transporter 5 from Polysciences). Two days posttransfection, cells were collected with 10 mM EDTA. Cells were stained with a 1:1,000 dilution of rabbit monoclonal or polyclonal anti–HNV-F (*SI Appendix*, Table S1) for 1 h at 4 °C, washed with 2% FBS in DPBS, stained with 1:2,000 dilution of anti–Rb-647 for 1 h at 4 °C, washed with 2% FBS in DPBS, and fixed with 2% PFA. All antibody dilutions used were optimized in pilot titration experiments to give the best signal:noise ratios using empty pCAGG vector-transfected 293T cells as the background (noise) control. Samples were washed and resuspended in 2% FBS in DPBS prior to being subjected to flow cytometry (Guava easyCyte). Multiple anti-HNV–F/G rabbit monoclonal and polyclonal antibodies were previously generated by various immunization strategies (*SI Appendix*, Table S1) (42, 43, 50, 75). Several polyclonal antibodies were tested against all of the HNV-F (WT and mutants) examined in this study. At least 2 polyclonal sera showed equivalent reactivity against WT NiV-F, WT HeV-F, and their cognate mutants (*SI Appendix*, Fig. S10). Therefore, mAb66, mAb36, and pAb835 binding was normalized to both of these pAbs (pAb2489 and pAb2490) to better account for any variation in the expression levels of the WT and mutant HNV-F proteins in this study.

### Pseudotyped Virus Production and Western Blot.

NiV or HeV F and G glycoproteins were pseudotyped by using VSV∆G-RLuc, a reporter virus in which the VSV-G glycoprotein has been replaced with a *Renilla* luciferase reporter gene. NiV and HeV pseudotyped particles (NiVpp and HeVpp) were prepared, as previously described (50, 76–78). Briefly, 293T cells were transfected to overexpress F and G glycoproteins, infected with VSV∆G-RLuc for 2 h, and then washed with DPBS. Two days postinfection, supernatant was collected, clarified by spinning at 1,250 rpm for 5 min, and purified by ultracentrifugation at 25,000 rpm for 2 h through a 20% sucrose cushion. NiVpp and HeVpp were resuspended in DPBS and aliquoted appropriately prior to storage at −80 °C to avoid multiple freeze–thaws.

For Western blot analyses of NiVpp and HeVpp incorporation of HNV glycoproteins (*SI Appendix*, Fig. S11), purified pseudotyped particles were lysed in Nonidet P-40 with 1× protease inhibitor, then mixed 6× Laemmli buffer and β-mercaptoethanol to a final concentration of 5%. Samples were then boiled for 10 min and run on a 4 to 15% Tris gradient gel prior to transfer onto a PVDF membrane. The membrane was stained sequentially with a 1:2,000 dilution of rabbit anti-AU1 (for F), rabbit anti-HA (for G), and mouse anti–VSV-M. Secondary antibodies, anti–Rb-647 or anti–Ms-647, were used at a 1:2,000 dilution.

### Neutralization Assay.

To titer NiVpp and HeVpp, 20,000 U-87 MG glioblastoma cells (ATCC HTB-14) were seeded in a 96-well plate and infected with a 10-fold serial dilution of pseudotyped particles. At 24 h postinfection, the cells were washed with DPBS, lysed and processed for the detection of *Renilla* luciferase activity by following the manufacturer’s instructions (Promega). Luminescence was read on the Cytation 3 (BioTek). From this titer, we determined the linear dynamic response range of our pseudotyped infection assay (*SI Appendix*, Fig. S12). A single viral dilution in that response range for each HNVpp sample was used and mixed with an equal volume of serially diluted monoclonal antibody. This mix was incubated at room temperature for 30 min, then used to infect U-87 MG cells for 24 h. The samples were processed for luciferase activity as described above.

### Detection of Diversifying Positive Selection (dN/dS Estimation).

Given that the diversity and number of sequences available for NiV is limited, we performed comparative analyses to detect diversifying positive selection within the fusion proteins of 2 paramyxoviruses that share similar structural and functional features with the NiV-F: MeV and PIV5. For this purpose, we generated complete and partitioned alignments corresponding to distinct protein domains of the NiV, MeV, and PIV5 fusion proteins, specifically group 1 (most distal region from viral membrane [DIII]), group 2 (middle region [DI, DII, and HRB linker]), group 3 (proximal region [HRB]), and group 4 (TM). The distribution of dN/dS ratios (ω) among sites was then estimated for each alignment using the M0/M1a/M2a and the M8/M8a site models in CODEML (56) (*SI Appendix*, *Supplementary Methods* and *Supplementary Results*). In addition, we used a Mann–Whitney *U* test to determine if the distribution of among-site ω values differed between 1) a model with a single ω distribution for all sites and 2) a model with 2 ω distributions, 1 for buried residues and 1 for surface-exposed residues.

In parallel, to extract maximum statistical power from our datasets of limited genetic variability, we performed a joint analysis that combined dataset-specific and region-specific effects on ω using a mixed-effect model (79). Under this model, ω can vary both across sites and branches using random-effects models, while the effect of the partition is fixed (79). We applied an unrestricted codon model of episodic diversification (79) to the complete and partitioned fusion protein alignments of NiV, MeV, and PIV5, and performed joint likelihood ratio tests to identify differences in selective pressure across codons within the different protein groups (*SI Appendix*, *Supplementary Methods* and *Supplementary Results*). For confirmatory purposes, additional tests were performed for all datasets using FUBAR (57).

### Data Deposition.

The atomic coordinates and structure factors have been deposited in the Protein Data Bank (PDB ID code 6T3F).

## Supplementary Material

Supplementary File
