# Supplementary material for: A structural basis for antibody-mediated neutralization of Nipah virus reveals a site of vulnerability at the fusion glycoprotein apex
Source: Proc Natl Acad Sci U S A. 2019 Nov 25;116(50):25057–67. doi: 10.1073/pnas.1912503116 (PMC6911215; doi:10.1073/pnas.1912503116)
Supplement: Supplementary File [file pnas.1912503116.sapp.pdf]

PNAS
www.pnas.org

Supplementary Information for

A structural basis for antibody-mediated neutralization of Nipah virus reveals a site of vulnerability at the fusion glycoprotein apex

Victoria A. Avanzato, Kasopefoluwa Y. Oguntuyo, Marina Escalera-Zamudio, Bernardo Gutierrez, Michael Golden, Sergei L. Kosakovsky Pond, Rhys Pryce, Thomas S. Walter, Jeffrey Seow, Katie J. Doores, Oliver G. Pybus, Vincent J. Munster, Benhur Lee, Thomas A. Bowden

Thomas A. Bowden
Email: thomas.bowden@strubi.ox.ac.uk

Benhur Lee
Email: benhur.lee@mssm.edu

This PDF file includes:

- Tables S1 to S4
- Figures S1 to S18
- SI Methods
- SI Results
- SI References

www.pnas.org/cgi/doi/10.1073/pnas.1912503116

Supplementary Table S1. Summary of monoclonal and polyclonal antibody features**.**

	Antibody	Immunogen (DNA Vaccination)	Binding[1]
**Monoclonal**	**mAb36**	HeV-F, HeV-G, NiV-M	HeV-F
	**mAb66**	NiV-F, G, NiV-M, soluble NiV-G	NiV and HeV-F
**Polyclonal**	**pAb835**	NiV-F, G and M	Anti-F
	**pAb1187**	Soluble NiV-G	Anti-G
	**pAb2489**	NiV-F, G, and M	Anti-F and Anti-G
	**pAb2490**	NiV-F, G, and M	Anti-F and Anti-G

*In blue are codon optimized constructs.
[1]Binding was quantified by flow cytometry, as described in the Materials and Methods.

Supplementary Table S2. Crystallographic data collection and refinement statistics.

	NiV-F Fab66
Data collection	
Beamline	I03
Wavelength (Å)	0.9763
Space group	P 63 2 2
Cell dimensions	
a, *b*, *c* (Å)	149.2, 149.2, 385.4
α, β, γ (°)	90, 90, 120
Resolution (Å)	107.34 - 3.20 (3.25 - 3.20)
R_{merge}	0.211 (2.231)
R_{meas}	0.215 (2.279)
R_{pim}	0.042 (0.456)
$CC_{1/2}$	0.998 (0.736)
$I/\sigma(I)$	13.9 (1.9)
Completeness (%)	99.6 (99.2)
Multiplicity	25.8 (24.3)
Unique reflections	42,722 (2,104)
Refinement	
Resolution (Å)	91.12 - 3.20 (3.31 – 3.20)
R_{work}/R_{free}	0.21/0.25
No. of atoms	
Protein	6,560
Ligand	84
B factors (Å^2)	
Protein	113.9
Ligands	142.9
Ramachandran	
Favored (%)	95.24
Allowed (%)	4.64
Outlier (%)	0.12%
RMS deviations	
Bond lengths (Å)	0.003
Bond angles (°)	0.605

[a]Values for the outer resolution shell are shown in parentheses.
[b]R_{free} is calculated as for R_{work}, using only 5% of the data separated prior to refinement.
[c]Ramachandran analysis determined with the Molprobity server (1).
[d]RMS deviations: root mean square deviation from ideal geometry.

Supplementary Table S3. Summary of the ω estimates for the paramyxoviral F protein and its functional subdomains (G1-4) using a maximum likelihood approach.

**MeV**	**ω ***	**PIV5**	**ω ***	**NiV**	**ω ***
Complete	0.1625<br>(0.1366 - 0.1915)	Complete	0.2623<br>(0.2034 - 0.3313)	Complete	0.0438<br>(0.0251 - 0.0699)
Group 1	DIII<br>0.147<br>(0.111 - 0.190)	Group 1	DIII<br>0.125<br>(0.072 - 0.199)	Group 1	DIII<br>0.0465<br>(0.0184 - 0.0943)
Group 2	DI, DII and HRB linker<br>0.109<br>(0.073 - 0.154)	Group 2	DI, DII and HRB linker<br>0.267<br>(0.170 - 0.394)	Group 2	DI, DII and HRB linker<br>0.0092<br>(0.0005 - 0.0405)
Group 3	HRB<br>0.207<br>(0.107 - 0.355)	Group 3	HRB<br>0.057<br>(0.003 - 0.253)	Group 3	HRB<br>0.0438<br>(0.0025 - 0.1947)
Group 4	TM<br>0.130<br>(0.071 - 0.214)	Group 4	TM<br>1.888<br>(0.904 - 3.404)	Group 4	TM<br>0.0000<br>(0.0000 - 0.0315)

[*]ω estimated under the MG94xREV model with all partitions analyzed jointly. Approximate 95% confidence intervals are based on profile likelihood.

Supplementary Table S4. Summary of mixed effects selection analyses.

Virus	Group	ω1 (weight)	ω2 (weight)	P-value for ω >1	P-value (among groups)
NiV-F	1	0.008 (1.00)	N.S	1	0.98
	2	0.000 (1.00)	N.S	1	0.98
	3	0.000 (1.00)	N.S	1	0.98
	4	0.000 (1.00)	N.S	1	0.98
PIV5-F	1	0.000 (0.977)	5.1 (0.023)	0.66	0.53
	2	0.224 (1.00)	N.S*	0.66	0.53
	3	0.059 (1.00)	N.S	0.66	0.53
	4	1.00 (0.132)	4.82 (0.867)	0.66	0.53
MeV-F	1	0.000 (0.987)	8.657 (0.013)	0.86	0.0018
	2	0.034 (1.00)	N.S	0.86	0.0018
	3	0.121 (1.00)	N.S	0.86	0.0018
	4	0.141 (1.00)	N.S	0.86	0.0018

**N.S = not supported by the data.*

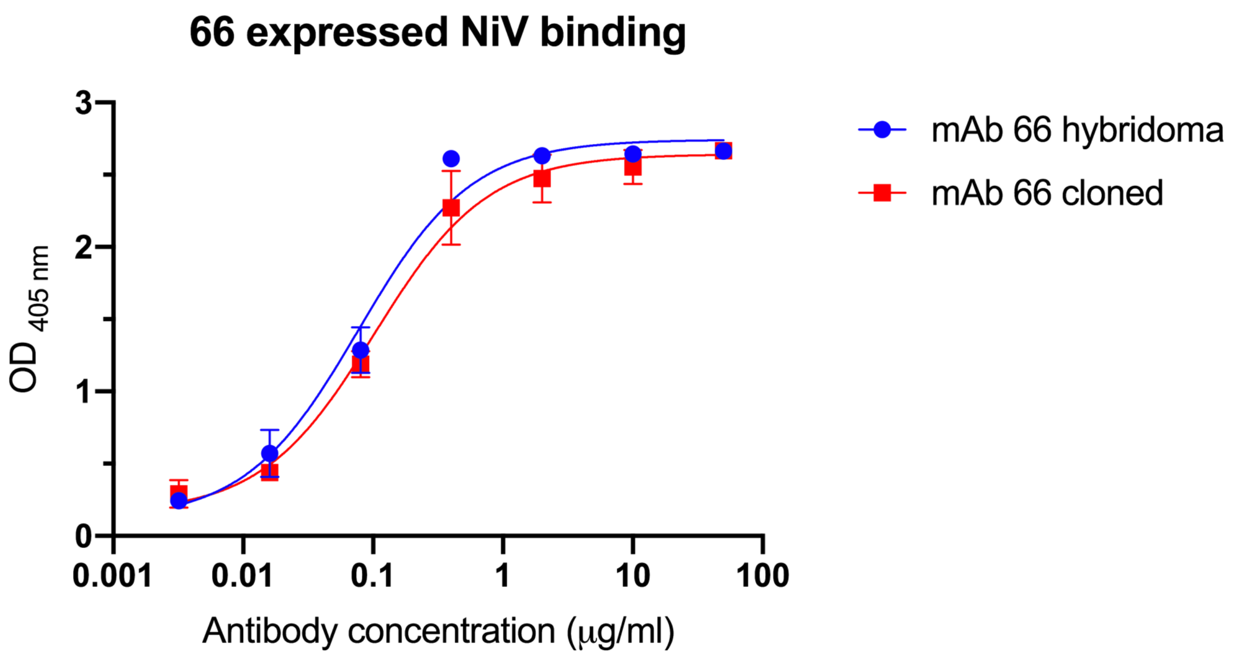

Fig. S1: ELISA comparing binding to recombinant soluble Nipah F of mAb66 purified from hybridoma supernatant (mAb66 hybridoma) and mAb66 expressed from the sequence rescued from the hybridoma cell-line (mAb66 cloned). These antibodies bind with similar apparent binding affinities.

A

	V Region	Identity (% nt)	J Region	Identity (% nt)	D Region	CDR1	CDR2	CDR3
66 Heavy	1S43*01	92.22	4*02	89.58	4-1*01	GFTLSSYW	LYTNGATT	ARGSGSGWSWFNI
66 Kappa	1S17*01	92.67	1-2*01	92.5		QNIISS	YAS	QSYYYSGITYGNA

B

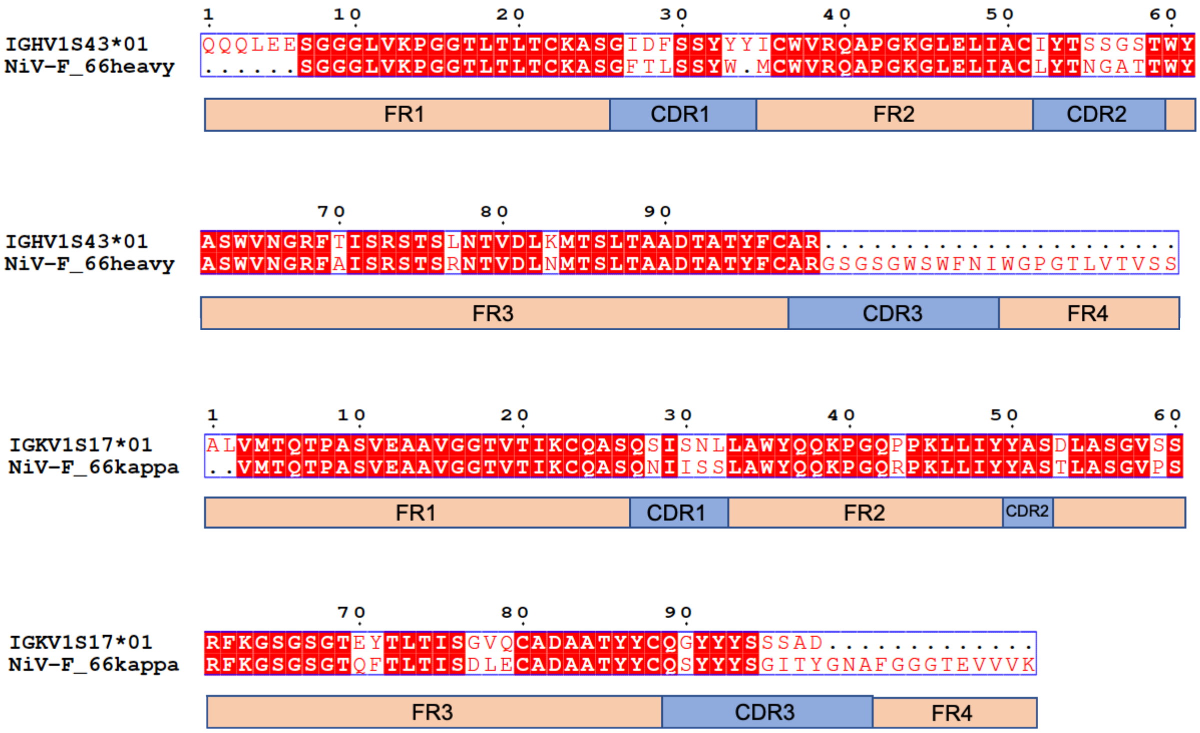

Fig. S2. Comparison of the rescued Fab66 sequence to the germline. (A) The international immunogenetics information system (IMGT) database (2, 3) was used to identify the most similar variable gene hits for both the heavy and kappa chain. (B) The Fab66 heavy and kappa sequences were aligned using Multalin (4) and displayed with ESPRIPT (5). CDR and framework regions are shown under the alignment.

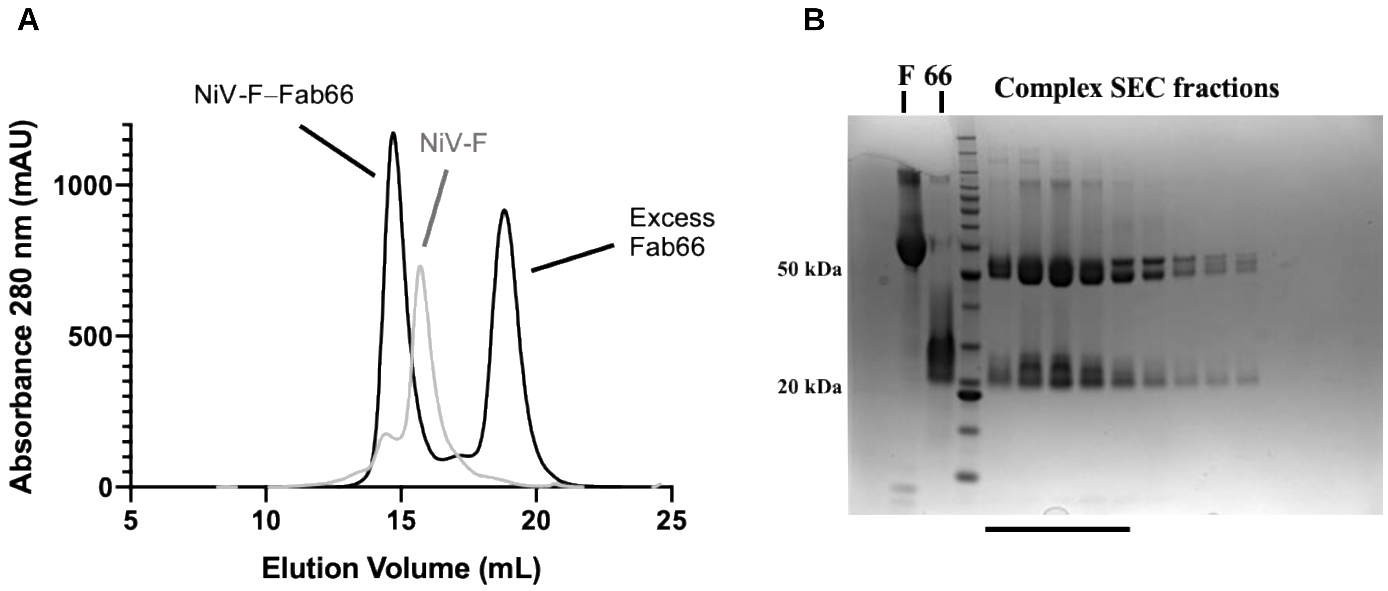

Fig. S3. NiV-F and Fab66 complex formation. (A) Overlay of size exclusion chromatography (SEC) profiles of NiV-F (grey) and NiV-F–Fab66 complex (black), prior to deglycosylation. A Superose 6 Increase 10/300 column equilibrated in 10 mM Tris pH 8, 150 mM NaCl was used for the purifications. (B) SDS-PAGE analysis. To the left of the marker, pre-endoglycosidase F_1-treated NiV-F (labelled 'F') and Fab66 (labelled '66') run under reducing conditions are shown. To the right of the marker, deglycosylated NiV-F–Fab66 complex SEC fractions run under reducing conditions are shown. Fractions pooled for crystallization are noted by a solid black line beneath the gel.

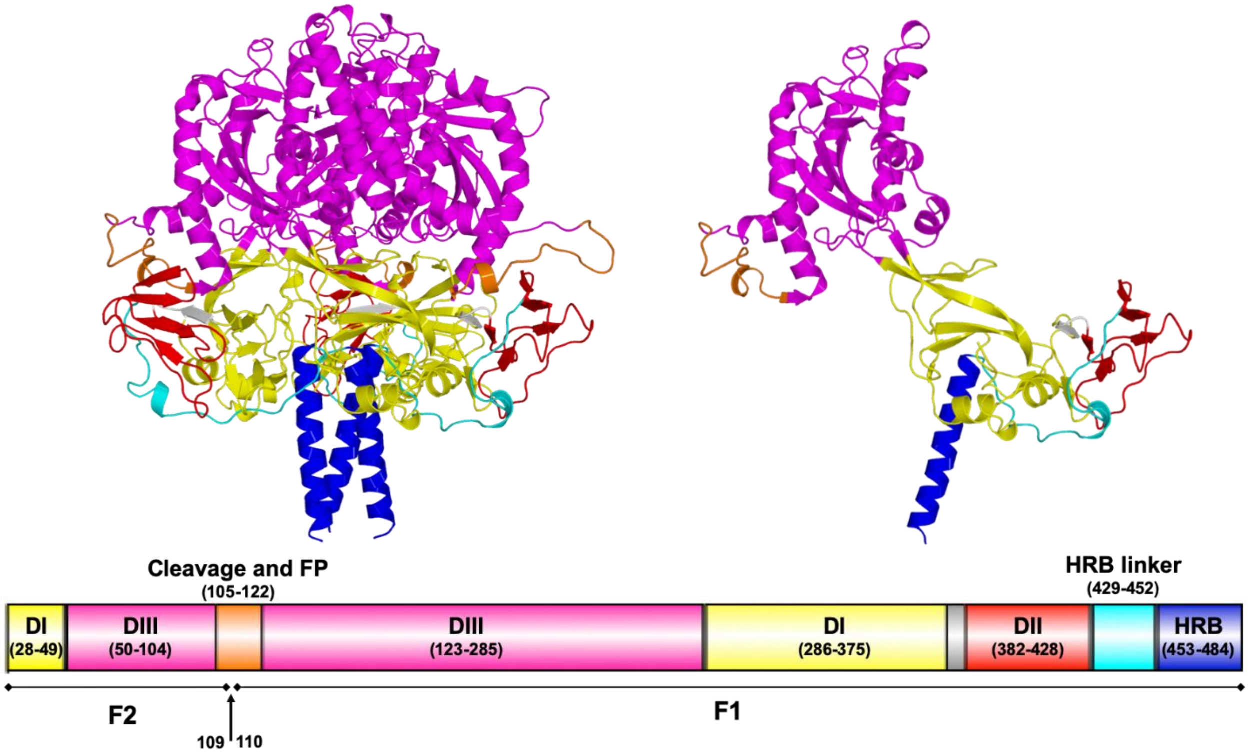

Fig. S4. Domain organization of the un-cleaved, prefusion, Fab66 bound NiV-F, produced using the DOG software (6). The Fab66-bound structure was organized into three domains, DI, DII, and DIII, followed by HRB linker and HRB. The residues comprising each domain were determined based off of the domain organization of the prefusion PIV5 fusion protein, as described previously (7). The cathepsin-L cleavage site R109–L110 is noted with a black arrow. The signal sequence precedes DI and the GCNt trimerization motif follows HRB (not shown).

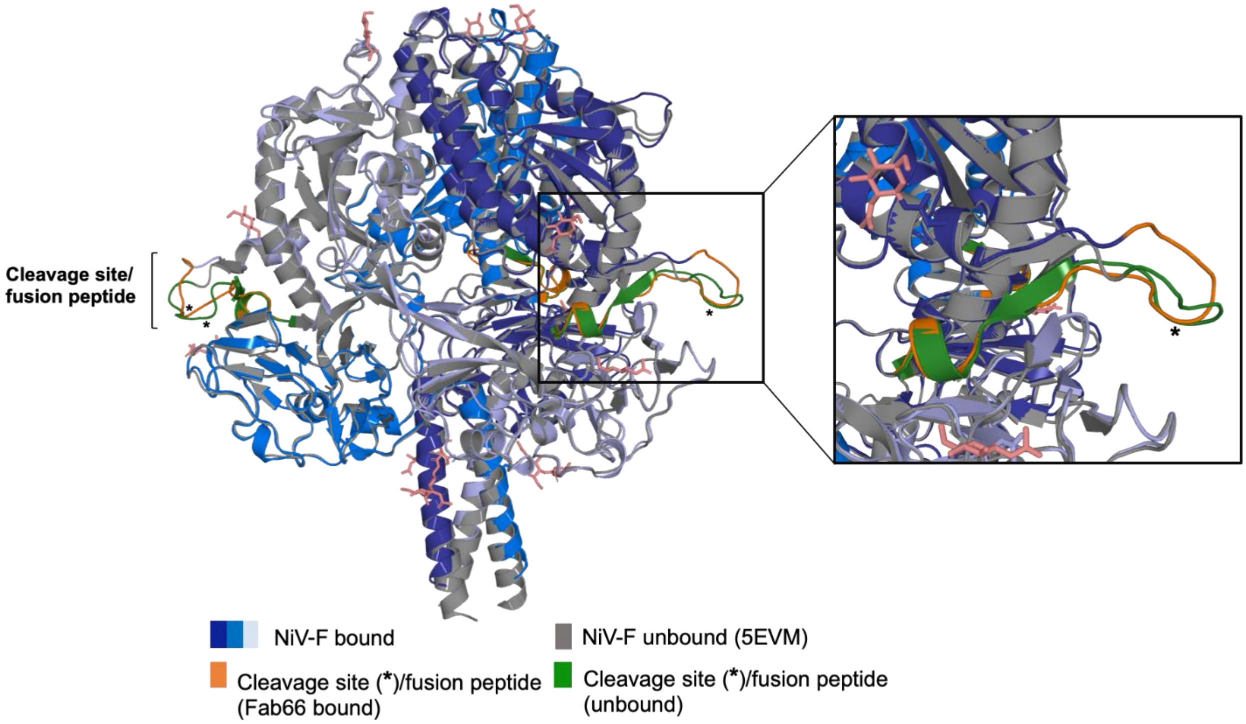

Fig. S5. Superposition of unbound and Fab66-bound NiV-F. The crystal structure of Fab66-bound NiV-F trimer is shown as cartoon and colored a different shade of blue for each protomer, and the unbound, un-cleaved, prefusion NiV-F (PDB ID 5EVM (8)) is rendered as cartoon and colored gray. The residues in the cleavage site and fusion peptide are colored green in the unbound structure and orange in the bound structure. The cathepsin-L cleavage site (R109–L110) is labeled with an asterisk (*). The two structures were aligned in COOT (9) using SSM superposition.

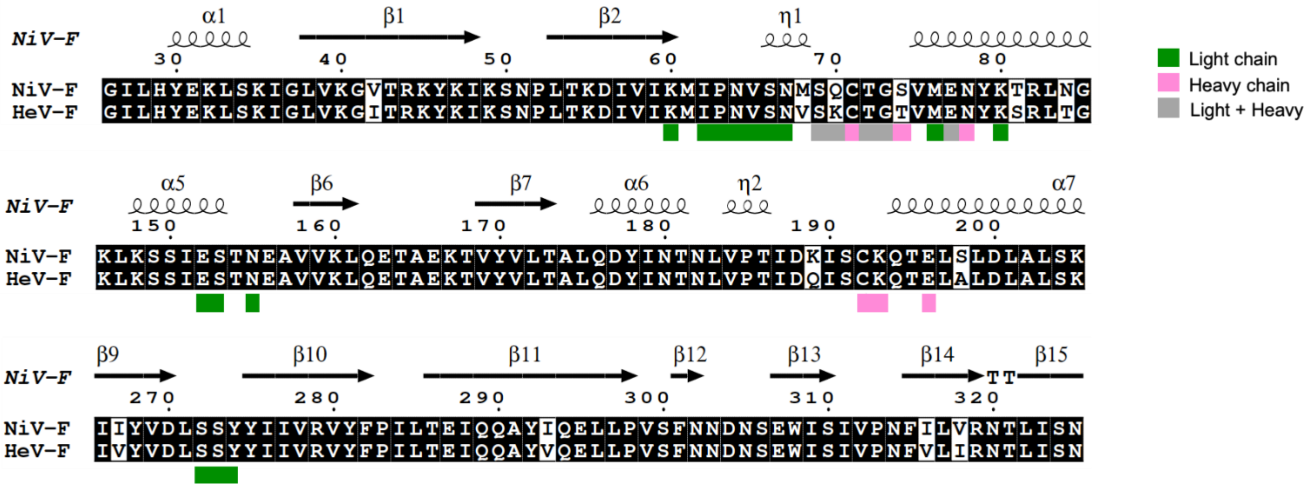

Fig. S6. Comparison of the Fab66 epitope on Nipah and Hendra fusion proteins. The binding footprint of Fab66 on the NiV-F surface (calculated by the PDBePISA server (10)) was mapped onto an amino acid sequence alignment of NiV-F (Malaysia, AAV80428.1) and HeV-F (AEB21197.1), generated by Multalin (4) and plotted with ESPript (5). Residues contacted by the light chain are noted by a green box below the sequence, residues contacted by the heavy chain with a pink box, and those contacted by both chains with a grey box. Secondary structure elements of the NiV-F protein (PDB 5EVM (8)) are shown above the alignment.

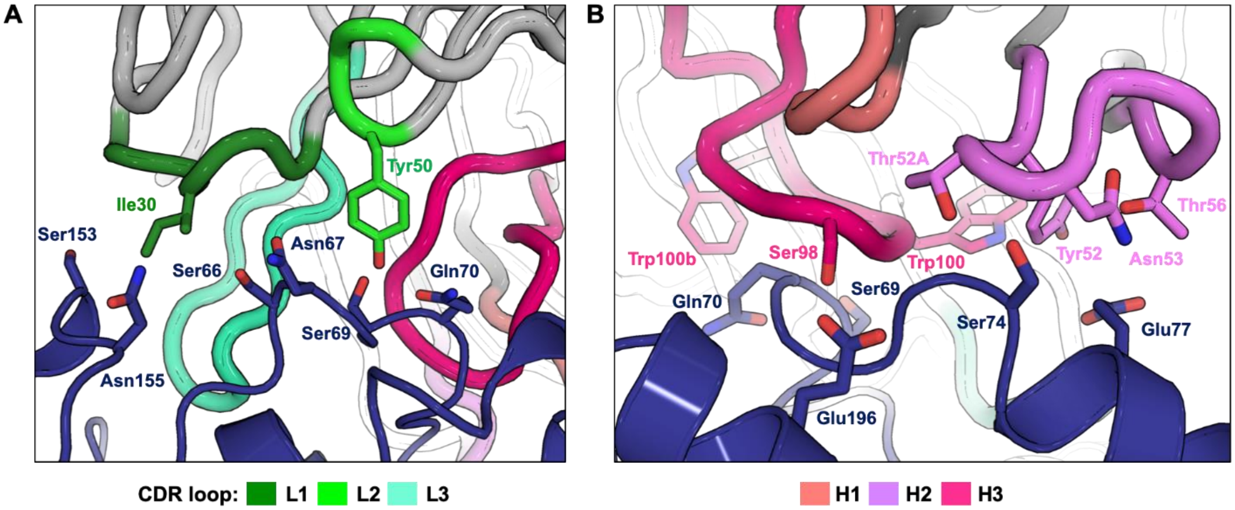

Fig. S7. Molecular interactions at the Fab66 – NiV-F interface. Fab66 is shown as a grey cartoon tube and NiV-F is shown as dark blue cartoon. CDR loops are colored in shades of pink (heavy chain) and green (light chain), as indicated. Highly buried residues and residues participating in intermolecular hydrogen bonds are shown as sticks. (A) CDR loops L1 and L2 make important contacts through residues Ile30 (dark green) and Tyr50 (light green), respectively. CDR L1 Ile30 forms a backbone hydrogen bond with residue Ser66 on NiV-F, and also heavily contacts Ser153 and Asn155. The sidechain of CDR L2 Tyr50 forms hydrogen bonds with residues Ser66, Ser69, and Gln70, and also contacts Asn67. (B) Heavy chain CDR H2 and H3 residues contribute to the interface. CDR H3 residue Ser98 forms sidechain and backbone contacts with NiV-F residues Gln70 and Glu196, while hydrophobic Trp100 contacts the sidechain and backbone of NiV-F residue Ser69. CDR H2 residues Thr52A and Asn53 hydrogen bond with NiV-F residue Ser74. Tyr52 and Thr56 from CDR H2 interact with additional residues Gly73 and Glu77 on NiV-F.

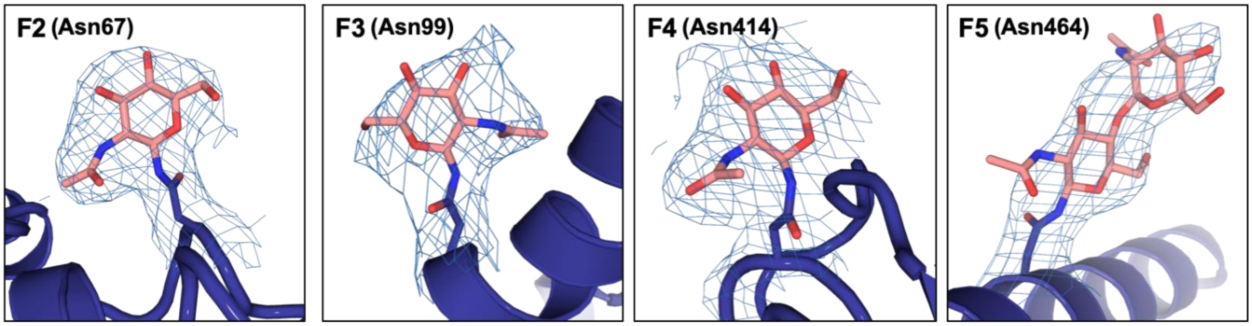

Fig. S8. *N*-linked glycosylation at sites F2–F5 NiV-F. Electron density (2*F*o–*F*c, 1.0 σ contour, light blue mesh) was observed and allowed modeling of the GlcNAc residues at the F2 (Asn67), F3 (Asn99), F4 (Asn414), and F5 (Asn464) glycan sites. No density was observed at the F1 (Asn64) glycan site.

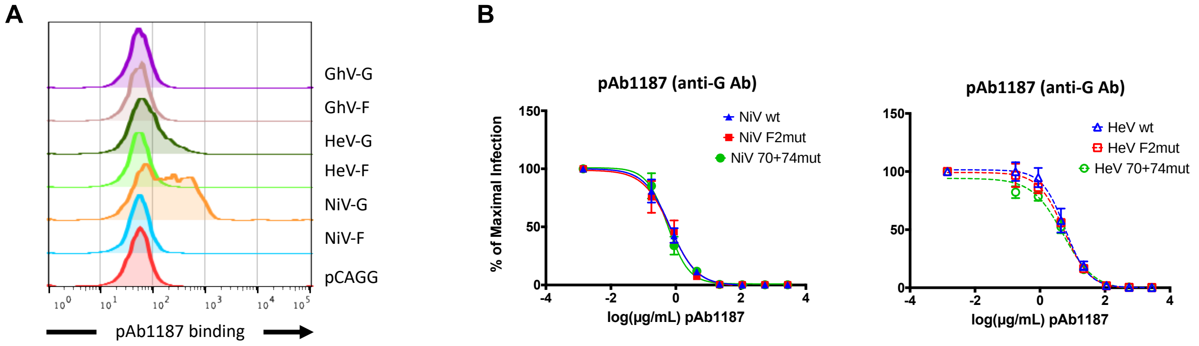

Fig. S9. Binding and neutralization of HNV-F and –G glycoproteins and mutants by G-specific polyclonal antibody. (A) Representative histogram plots of 1:1,000 dilution of pAb1187 binding to HNV-F or -G glycoproteins. Binding was performed as described in the Methods and analyzed as described in **Fig. 3D**. Ghana virus F and G glycoproteins (GhV-F and GhV-G) were used as negative and specificity controls as they are from a highly divergent African henipavirus previously determined to have limited to no antigenic relationship to the prototypical HeV and NiV found in Indian, Bangladesh, and Southeast Asia (11). (B) As a control, neutralizations were performed with an anti-G specific polyclonal and reveal no differences in neutralization of the wt and mutant F constructs. Neutralization curves of NiVpp and HeVpp (left and right panels, respectively) infection using wild type, F2mut, and the cognate 70+74mut, were generated and analyzed as described in **Fig. 3D**. Each curve was performed in biological triplicates comprised of technical duplicates per biological replicate. Data points shown are mean +/- S.E. Statistical significance was determined as described in in **Fig. 3D** legend (*, $p \leq 0.05$; **, $p \leq 0.01$; ***, $p \leq 0.001$; ****, $p \leq 0.0001$).

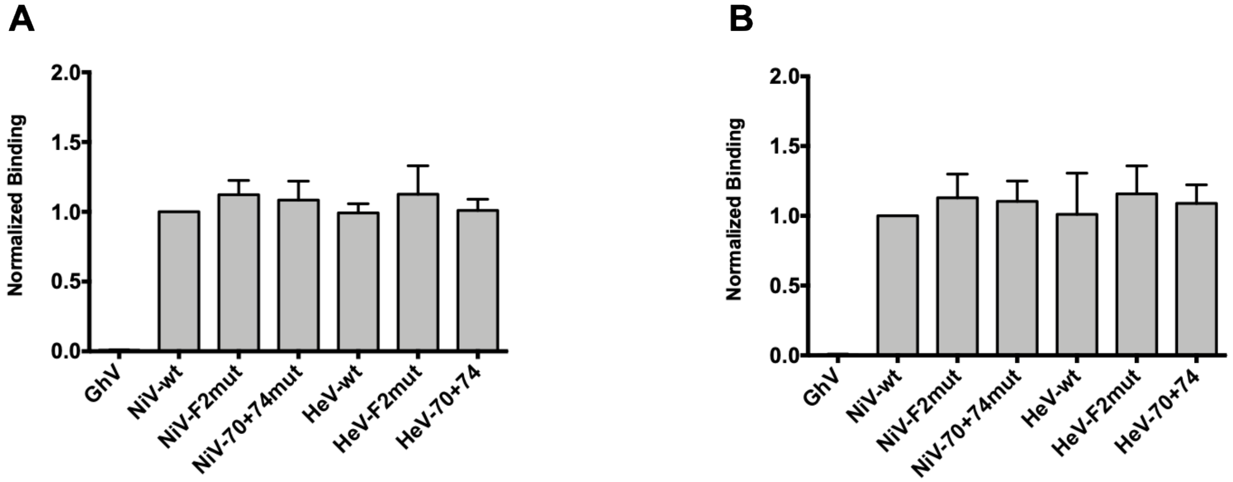

Fig. S10. pAb2489 and pAb2490 recognize wild type and mutant NiV-F or HeV-F glycoproteins similarly. Wt HNV-F and the indicated mutants were transfected into 293T cells and detected by pAb2489 (A) or pAb2490 (B) as described in Methods. GhV-F was used as a negative control. The GMFI of all the indicated constructs were normalized to the GMFI value of wt NiV-F, which was set at 1. Data shown as mean +/- S.E. from 3 independent experiments. Compared to wt NiV-F, none of the mutant NiV-F or HeV-F constructs showed any statistically significant differential binding to either pAb2489 or pAb2490 (ordinary one-way ANOVA with Dunnett's correction for multiple comparisons). Note, these pAb sera were generated using codon-optimized NiV-F, -G and-M (**Table S1**) and contain variably cross-reactive antibodies against both HNV-F and G glycoproteins. The polyclonal response is spread over both F and G, which likely dilutes immune focusing on any particular region of F and G. In any case, the data show that these antibodies are valid for use in normalizing the binding of other Abs that may be affected by the indicated HNV-F mutations. In contrast, pAb835 was generated with codon-optimized NiV-F, but wt NiV-G and NiV-M. Our wt NiV-G is expressed at much lower levels from the pcDNA3 vector we were using at the time. Thus, our immunization scheme that elicited pAb835 resulted in an anti-NiV-F-specific polyclonal response (**Table S1** and last subsection of results section) that is

likely focused on the immunologically accessible regions posited in the model presented in this study.

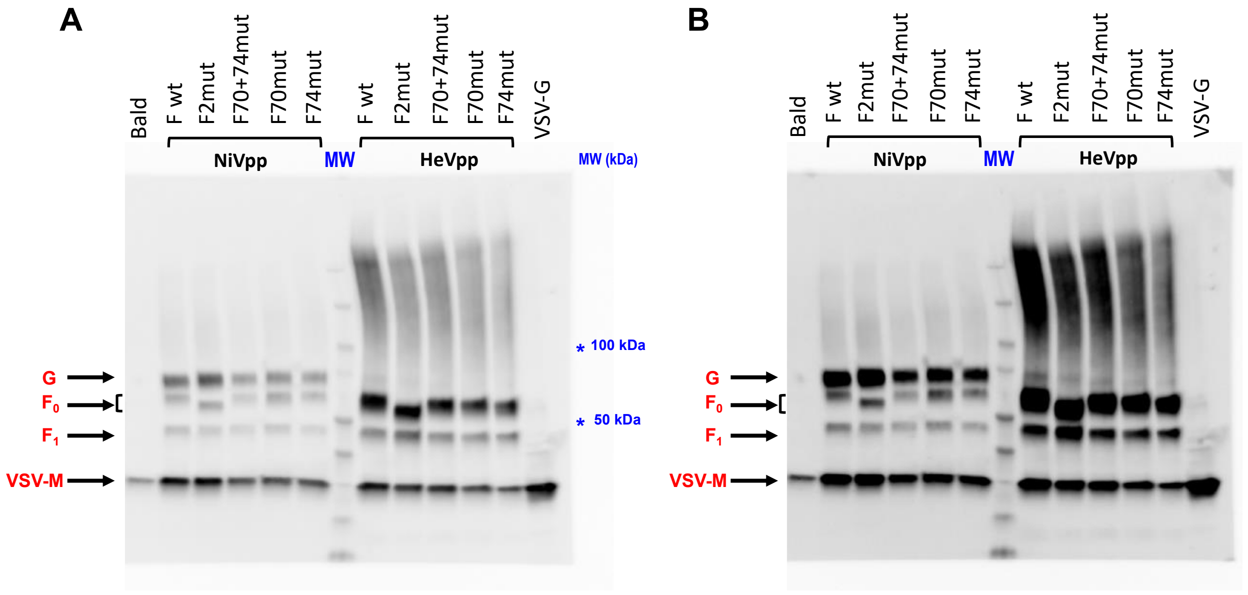

Fig. S11. NiV and HeV virus F and G protein incorporation into VSV-ΔG-RLuc pseudotyped particles (NiVpp and HeVpp). Purified NiVpp and HeVpp were prepared, as described in the Methods. (A) Equivalent protein amounts (quantified by Bradford assay) of the indicated HNVpp stocks were loaded into each lane of a 4-15% SDS-Tris gradient gel and subsequently subjected to sequential western blotting for detection of HNV-F (rabbit anti-AU1 C-terminus tag), HNV-G (rabbit anti-HA C-terminus tag), and VSV-M (mouse anti-VSV-Matrix). The latter served as a loading control. (B) The same gel was overexposed to reveal the weaker signal intensity of the HeV-G band. The removal of the F2 N-glycan site (F2mut) is evidenced by the faster mobility of the F_0 band in the F2mut lane for both NiVpp and HeVpp (A and B). The F_1 band does not exhibit any change in mobility as the F2 N-glycan site is in the smaller cleaved F_2 subunit, which is not detected by the anti-AU1 C-terminal tag.

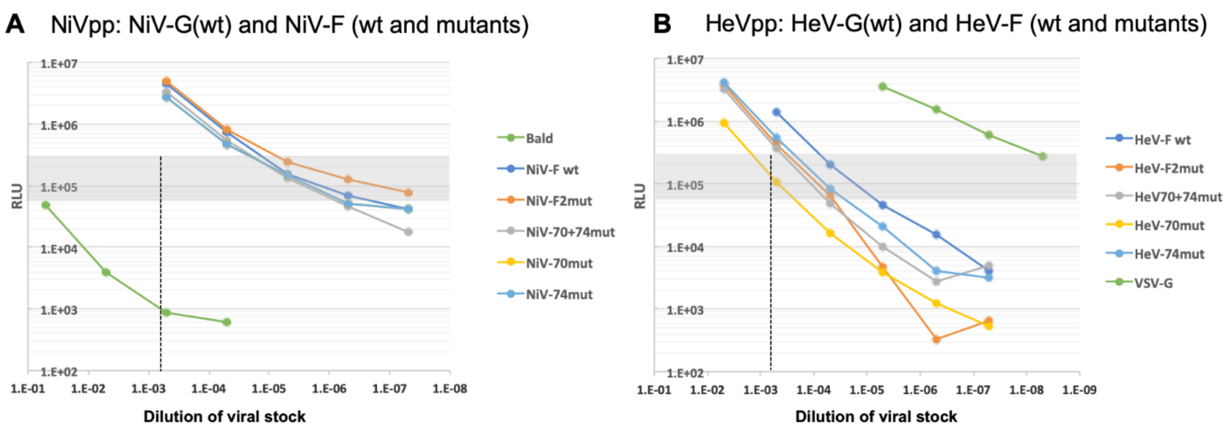

Fig. S12. NiVpp and HeVpp wild-type and F-mutant titers on U87 cells. Purified (A) NiVpp and (B) HeVpp containing wt HNV-G and the indicated wt or mutant F proteins were titered on highly permissive U87 glioblastoma cells via 10-fold serial dilutions of the concentrated viral stock in a 96-well format. To ensure valid comparisons in antibody neutralization experiments, we aimed for viral inputs will give ~10^5 RLU (relative light units) per 96-well, which is within the dynamic response range of the assay for all the mutants tested (shaded grey in A and B). Since there is also inherent background arising from the remnant VSV-G used to pseudotype the VSVpp[ΔG-RLuc] stock used to prepare these HNVpp—green "Bald" particles in (A)—we also aimed for a viral input where the background from any remnant VSV-G is minimal (<10^{-3} dilution of virus stock, vertical dotted line in A and B). The VSV-G pseudotyped particles, green "VSV-G" particles in (B), serves as a positive control when titering any new HNVpp stock.

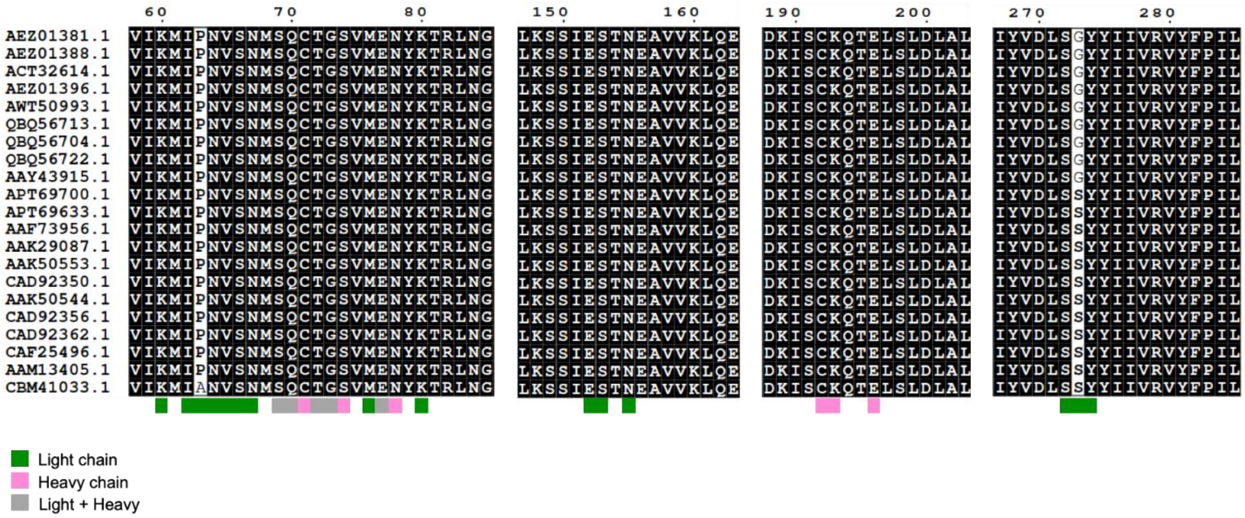

Fig. S13. The Fab66 epitope is highly conserved among NiV virus strains. Available NiV-F protein sequences were aligned using Multalin (4) and plotted using ESPript (5). The Fab66 epitope (calculated by the PDBePISA server (10)) was mapped onto the alignment; residues contacted by the light chain are noted by a green box below the sequence, the residues contacted by the heavy chain with a pink box, and those contacted by both chains with a grey box.

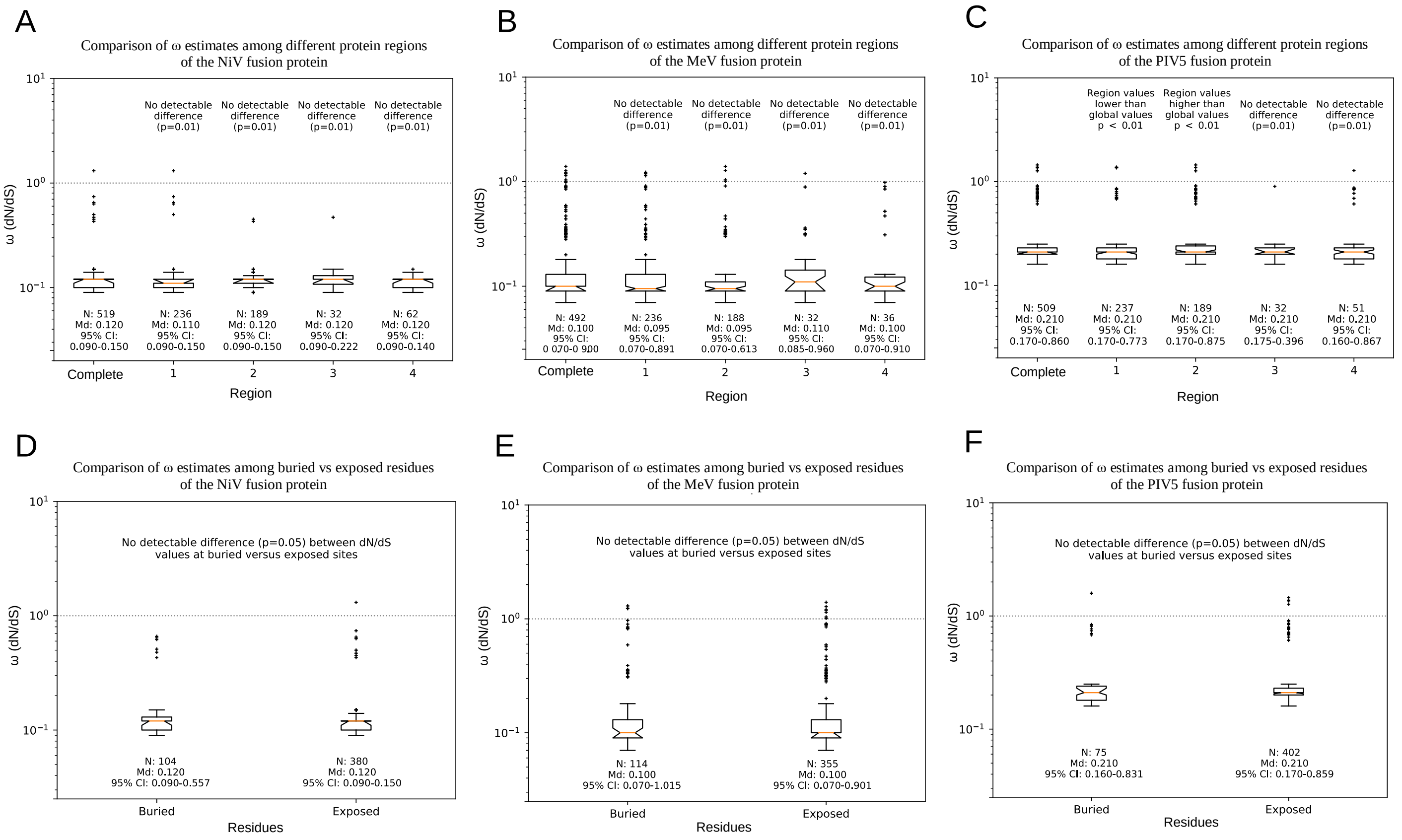

Fig. S14. dN/dS estimation of the NiV, MeV, and PIV5 fusion proteins using a maximum likelihood approach. (A–C) ω estimation for the entire fusion protein coding region, as well as different regions of the protein, were assessed for diversifying positive selection. Group 1 = domain III, most distal from viral membrane, Group 2 = DI, DII, and HRB linker, Group 3 = membrane proximal stalk and HRB, Group 4 = transmembrane domain. (D–F) ω estimation for buried versus solvent exposed residues.

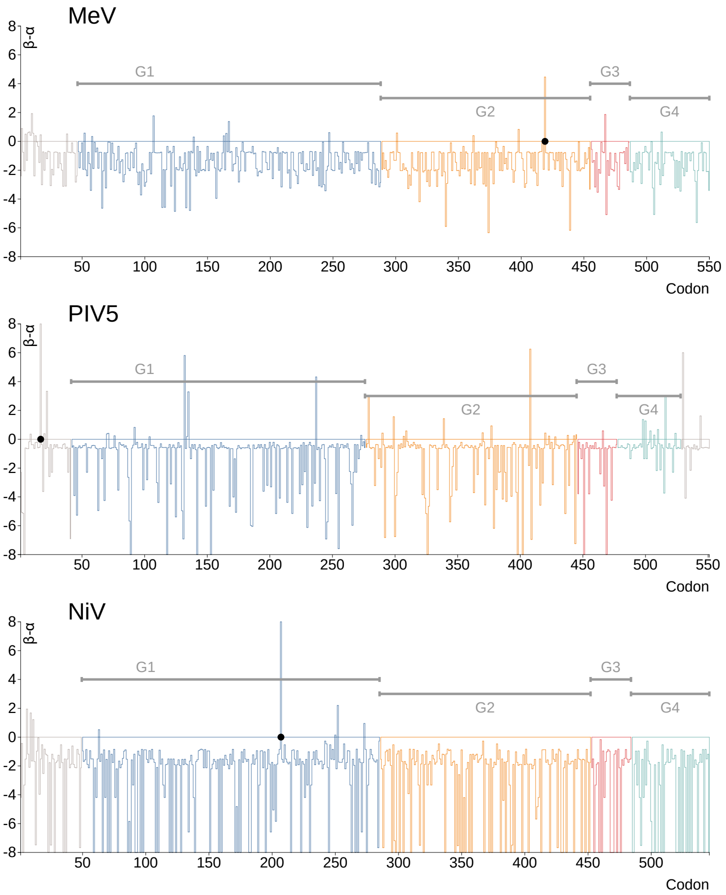

Fig S15. Plots for the ω values across sites of the F protein of different paramyxoviruses (MeV, PIV5 and NiV), representing the estimates of site-level difference in non-synonymous and synonymous substitution rates (β-α, equivalent to ω) across the complete protein and within different functional domains (G1-4), as obtained with the FUBAR method (negative selection < 0, neutrality = 0, positive selection >1). Sites where posterior probability scoring for diversifying positive selection (β-α > 0) exceeded 0.9 are shown with dots.

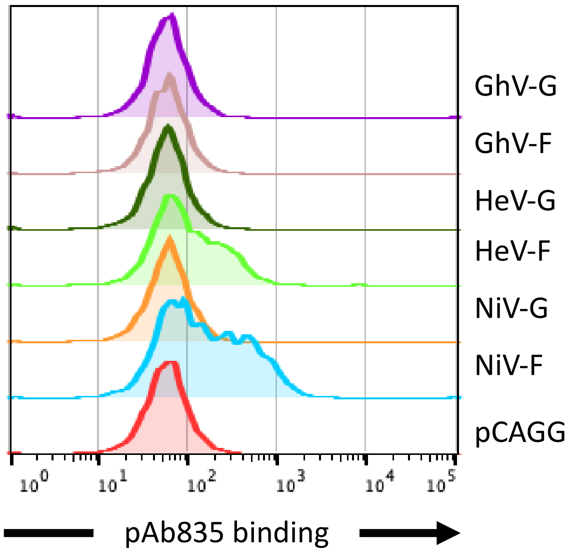

Fig. S16. Binding of HNV-F and G glycoproteins by F-specific rabbit polyclonal antibodies. (A) Representative histogram plots of 1:1,000 dilution of pAb835 binding to HNV-F or -G glycoproteins. Binding was performed as described in the methods and analyzed as described in **Fig. 3D**. Ghana virus F and G glycoproteins (GhV-F and GhV-G) were used as negative and specificity controls as they are from a highly divergent African henipavirus previously determined to have limited to no antigenic relationship to the prototypical HeV and NiV found in Indian, Bangladesh and Southeast Asia (11).

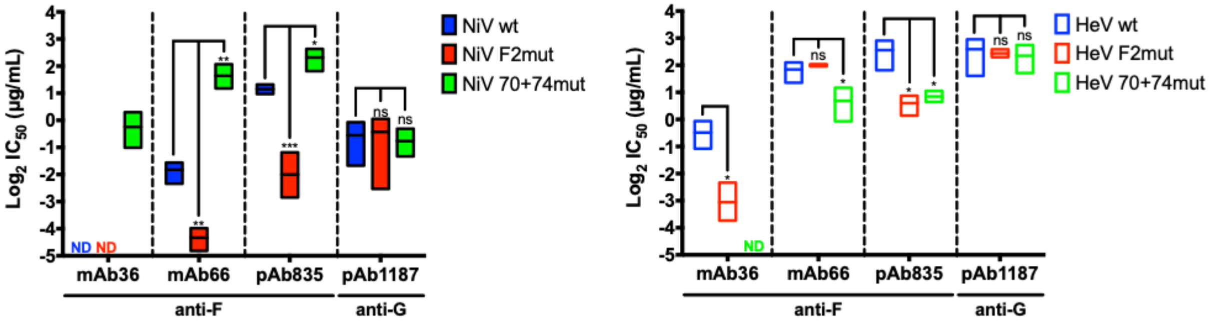

Fig. S17. Summary of neutralization data for all monoclonal and polyclonal antibodies with distinct HNV-F or -G specificities. IC_{50} values of neutralization curves for all the antibodies tested against wt or mutant HNV-F bearing NiVpp (left panel) and HeVpp (right panel) are summarized here as box plots for ease of comparison. Each box plot shows the range (minimum to maximum) and mean (horizontal line) IC_{50} values from neutralization curves generated from three biological replicates (as described in **Fig. 3D**). ND for mAb36 denotes 'not determined' due to ambiguous or no fit (representing no neutralization for HNVpp bearing the indicated HNV-F proteins, **Fig. 6A** and **B**). Statistical significance was determined by one sample t-Test (*, $p \leq 0.05$; **, $p \leq 0.01$; ***, $p \leq 0.001$).

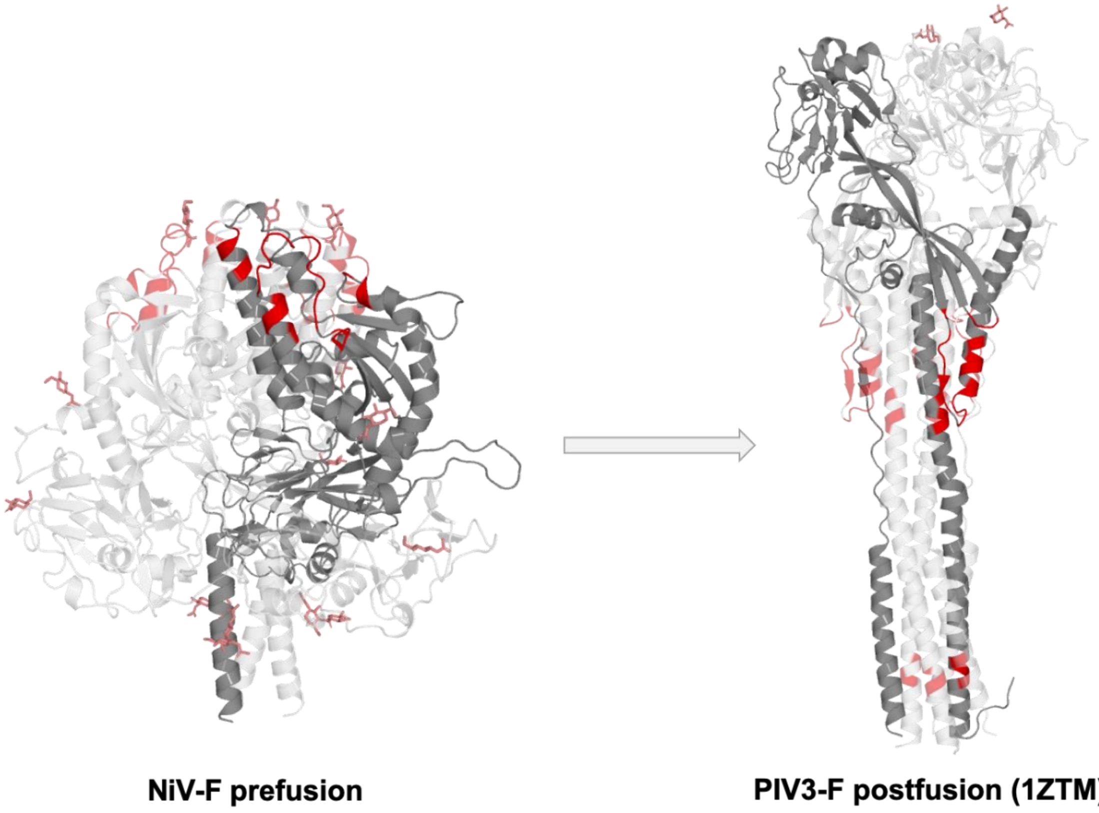

Fig. S18. The Fab66 epitope becomes disrupted in the expected postfusion conformation. NiV-F prefusion and PIV3-F postfusion (PDB ID 1ZTM (12)) are shown as cartoon and colored grey, with one protomer in the trimer colored a darker shade of gray for clarity. Residues contacted by Fab66 (as identified by the PDBePISA server (10) are colored red. The Fab66 epitope was mapped onto the postfusion structure of the PIV3-F via a sequence alignment, and the equivalent residues were colored red on the postfusion structure.

Supplementary Methods for dN/dS (ω) analyses

CODEML. All available complete coding sequences for the NiV, MeV and PIV5 fusion proteins, from all hosts and geographical regions, were retrieved from the NCBI GenBank database as of May 2019. 56 sequences, 26 sequences, and 18 sequences were retrieved and downloaded for MeV, PiV5, and NiV, respectively. As a measure of diversity within the complete alignments, the overall mean distance (number of base substitutions per site averaging over all sequences) was calculated using the Kimura 2 parameter model (13). An overall mean distance of 0.039 for NiV-F, of 0.036 for MeV-F, and of 0.022 PIV5-F was estimated, indicating an overall low diversity within all alignments. Individual datasets were aligned using MAFFT v7 and maximum likelihood phylogenies were estimated using RAxML under the GTR+G model and bootstrapping with 100 replicates (14). Partitions were derived from the global alignments, corresponding to distinct protein domains (Group 1-4, as defined in the main text). Global and site-specific ω values were estimated under M0/M1a/M2a and the M8/M8a site models using CODEML in PAML (15), and confirmed using the FUBAR method (16). The statistical significance of nested models was evaluated using a likelihood ratio test (LRT), using the $\chi 2$ approximation to the likelihood ratio. Only positively selected sites (PSS) scored under Bayes Empirical Bayes (BEB), or/under a posterior probability above 0.9 were considered (15, 16). The null hypothesis of neutral or negative selection only is explicitly included in the analysis as a statistical "control" under a LTR. Within the *per site* analyses under the CODEML (M8) and FUBAR methods, all regions of the protein (including the DIII apex tip and the Fab66 binding epitope) were considered. Based on this *per site* analyses, specific regions of interest of the NiV-F (the apex region and the complete Fab66 epitope) were further analyzed to estimate mean ω values using an approximation to a normal distribution to calculate 95% confidence intervals. The region for the apex tip included residues:

Val58−Gly85, Ala141−Ser208, Ala234−Leu246, and Asp270−Tyr275. The region for the Fab66 epitope included residues: Lys60, Ile62−Asn67, Ser69−Ser74, Met76−Asn78, Lys80, Glu152−Ser153, Asn155, Cys192−Lys193, Glu196, and Ser272−Tyr274.

Mixed effect model. In order to extract maximum statistical power from small/low divergence datasets, a joint analysis which combines a region effect with a dataset effect on ω values, *e.g.* ω *= function (dataset, region)* was performed for all fusion protein alignments. We used an unrestricted codon model of episodic diversification (17), in which ω at each branch and site of a given partition, **p = 1.4,** is modelled from a 2-bin general discrete distribution, with $0 \leq \omega_1(\mathbf{p}) \leq 1 \leq \omega_2(\mathbf{p})$, and a probability of $(\omega = \omega_1 (\mathbf{p})) = f(\mathbf{p})$. Consequently, ω can vary both across sites and branches using random effects models, while the effect of the partition is fixed. Because viral sequences isolated from individual hosts may harbour many neutral or even deleterious mutations (at the population level) due to within-host evolution dynamics (18, 19), the evolution along terminal branches of the tree is modelled with separate (nuisance) distributions, and the focus of interference are the parameters governing evolution along internal tree branches. These distribution parameters, together with other model parameters (branch lengths, nucleotide substitution biases, nuisance terminal branch parameters) are estimated jointly from all partitions using maximum likelihood. We further assumed that branch lengths are shared by all partitions up to a per-partition scaling factor (the relative ratio model of evolution). In this model, two hypotheses are tested under a LRT:

1. *Assess if there are any partitions subject to positive selection.* This is done by comparing the full model, described above, with the null model where, for each **p**,

ω_2(**p**) is constrained to be 1, and assessing significance using the likelihood ratio test. P-values are derived from the χ^2 distribution with 4 degrees of freedom.

2. *Assess if selective regimes differ between partitions.* This is done by comparing the full model, with the null model where, for each **p>1**, ω_1(**p**) = ω_1(**1**), ω_2(**p**) = ω_2(**1**), and f(**p**) = f(**1**), and assessing significance using the likelihood ratio test. P-values are derived from the χ^2 distribution with 9 degrees of freedom.

The testing procedure is implemented as a script in HyPhy v2.5 (20) and is available from https://github.com/veg/hyphy-analyses/tree/master/PartitionedSelection

Supplementary Results for dN/dS (ω) analyses. No evidence for positive selection (ω >1) upon the complete coding region or per functional group within the F protein, including the apex of DIII and the Fab66 binding epitope, was detected for any of the paramyxoviruses analyzed (**Fig. S14** and **S15**). No positively selected sites were detected under the M2a and M8 site models, as were significantly scored under BEB (**Table S3**). For the MeV-F domain analysis, no significant differences were observed amongst the four different functional groups compared to a global ω estimate, and no significant differences were observed between the ω estimates for buried vs. solvent exposed residues (**Fig. S14B** and **E**, **Table S3**). A similar pattern was observed for NiV-F, with no significant differences amongst the four different group regions, and across the ω estimates for buried vs. solvent exposed residues (**Fig. S14A** and **D**, **Table S3**). For the PIV5 fusion protein, the ω estimate for Group 1 was marginally lower compared to all other regions, whereas the ω estimate for Group 2 were marginally higher compared to all other regions, and no differences were observed between the ω estimates for buried vs. exposed residues (**Fig. S14C** and **F**, **Table S3**). ML analysis under M8 revealed that the highest proportion of sites for each

alignment are under the strong conservation class, corresponding to purifying (negative) selection (ω << 1): MeV p: 0.20000, ω: 0.00000; for NiV p: 0.19950, ω: 0.01561; for PIV5 p: 0.19475, ω: 0.00000. FUBAR analysis also revealed a similar pattern at the site level (**Fig. S15**). Of the 219 categorizable sites (out of 550) under a posterior probability of > 0.9 for MeV-F, 218 show evidence for negative selection (i.e. under selective constraints) and only 1 exhibits evidence for positive selection (i.e. under putative adaptive selective pressure). Of the 93 categorizable sites (out of 546) under a posterior probability of > 0.9 for NiV-F (many of which are included in DIII), 92 exhibit evidence of negative selection and only 1 exhibits evidence for positive selection. Of the 9 categorizable sites (out of 551) under a posterior probability of > 0.9 for PIV5, 8 exhibit evidence for negative selection, and only 1 exhibits evidence for positive selection. These results provide direct evidence of purifying negative selection across the majority of sites ranked under significance, supporting the hypothesis that strong functional constraints on the paramyxoviral F protein. Comparative methods are not able to infer the nature of selection on perfectly conserved sites, as they require substitutions to gain power. Therefore, if one considers perfectly conserved sites as being under strong purifying selection, this only strengthens our hypothesis.

A mixed effects model analysis revealed a strong and consistent degree of conservation across all codon groups for all virus datasets analyzed, confirming our previous observations. For NiV, an ω of 0 or near zero was obtained along internal branches in viral phylogeny, with no evidence for episodic positive selection anywhere in the tree (**Table S4**). The evolutionary patterns observed for PIV and MeV, with Group 1 and 4 (in PIV) and Group 1 (in MeV) yielded non-zero estimates for a fraction of branches and sites evolving with an ω >1. However, there was insufficient statistical support for positive selection acting upon any of the partitions tested. The

only significant difference was observed for MeV, driven by differences in ω distributions between G1 and other functional groups (**Table S4**).

Supplemental References

1. Chen VB, *et al.* (2010) MolProbity: all-atom structure validation for macromolecular crystallography. *Acta Crystallogr D Biol Crystallogr* 66(Pt 1):12-21.
2. Giudicelli V, Brochet X, & Lefranc MP (2011) IMGT/V-QUEST: IMGT standardized analysis of the immunoglobulin (IG) and T cell receptor (TR) nucleotide sequences. *Cold Spring Harb Protoc* 2011(6):695-715.
3. Brochet X, Lefranc MP, & Giudicelli V (2008) IMGT/V-QUEST: the highly customized and integrated system for IG and TR standardized V-J and V-D-J sequence analysis. *Nucleic Acids Res* 36(Web Server issue):W503-508.
4. Corpet F (1988) Multiple sequence alignment with hierarchical clustering. *Nucleic Acids Res* 16(22):10881-10890.
5. Robert X & Gouet P (2014) Deciphering key features in protein structures with the new ENDscript server. *Nucleic acids research* 42(Web Server issue):W320-324.
6. Ren J, *et al.* (2009) DOG 1.0: illustrator of protein domain structures. *Cell Res* 19(2):271-273.
7. Yin HS, Wen X, Paterson RG, Lamb RA, & Jardetzky TS (2006) Structure of the parainfluenza virus 5 F protein in its metastable, prefusion conformation. *Nature* 439(7072):38-44.
8. Xu K, *et al.* (2015) Crystal Structure of the Pre-fusion Nipah Virus Fusion Glycoprotein Reveals a Novel Hexamer-of-Trimers Assembly. *PLoS Pathog* 11(12):e1005322.
9. Emsley P, Lohkamp B, Scott WG, & Cowtan K (2010) Features and development of Coot. *Acta Crystallogr D Biol Crystallogr* 66(Pt 4):486-501.
10. Krissinel E & Henrick K (2007) Inference of macromolecular assemblies from crystalline state. *J Mol Biol* 372(3):774-797.
11. Lee B, *et al.* (2015) Molecular recognition of human ephrinB2 cell surface receptor by an emergent African henipavirus. *Proc Natl Acad Sci U S A* 112(17):E2156-2165.
12. Yin HS, Paterson RG, Wen X, Lamb RA, & Jardetzky TS (2005) Structure of the uncleaved ectodomain of the paramyxovirus (hPIV3) fusion protein. *Proc Natl Acad Sci U S A* 102(26):9288-9293.
13. Kumar S, Stecher G, & Tamura K (2016) MEGA7: Molecular Evolutionary Genetics Analysis Version 7.0 for Bigger Datasets. *Mol Biol Evol* 33(7):1870-1874.
14. Stamatakis A (2014) RAxML version 8: a tool for phylogenetic analysis and post-analysis of large phylogenies. *Bioinformatics* 30(9):1312-1313.
15. Yang Z (2007) PAML 4: phylogenetic analysis by maximum likelihood. *Mol Biol Evol* 24(8):1586-1591.
16. Murrell B, *et al.* (2013) FUBAR: a fast, unconstrained bayesian approximation for inferring selection. *Mol Biol Evol* 30(5):1196-1205.
17. Murrell B, *et al.* (2015) Gene-wide identification of episodic selection. *Mol Biol Evol* 32(5):1365-1371.
18. Pond SL, *et al.* (2006) Adaptation to different human populations by HIV-1 revealed by codon-based analyses. *PLoS Comput Biol* 2(6):e62.

19. Pybus OG, *et al.* (2007) Phylogenetic evidence for deleterious mutation load in RNA viruses and its contribution to viral evolution. *Mol Biol Evol* 24(3):845-852.
20. Pond SLK, Frost SDW, & Muse SV (2005) HyPhy: hypothesis testing using phylogenies. *Bioinformatics* 21(5):676-679.
